# Comparative Phosphoproteomic Profiling of Type III Adenylyl Cyclase Knockout and Control, Male, and Female Mice

**DOI:** 10.3389/fncel.2019.00034

**Published:** 2019-02-13

**Authors:** Yuxin Zhou, Liyan Qiu, Ashley Sterpka, Haiying Wang, Feixia Chu, Xuanmao Chen

**Affiliations:** ^1^Department of Molecular, Cellular and Biomedical Sciences, University of New Hampshire, Durham, NH, United States; ^2^Department of Statistics, University of Connecticut, Storrs, CT, United States

**Keywords:** Type III adenylyl cyclase (AC3), primary cilia, major depressive disorder (MDD), autistic spectrum disorder (ASD), phosphoproteomics, delta catenin, sexual dimorphism of autism, gender-biased phosphorylation

## Abstract

Type III adenylyl cyclase (AC3, *ADCY3*) is predominantly enriched in neuronal primary cilia throughout the central nervous system (CNS). Genome-wide association studies in humans have associated *ADCY3* with major depressive disorder and autistic spectrum disorder, both of which exhibit sexual dimorphism. To date, it is unclear how AC3 affects protein phosphorylation and signal networks in central neurons, and what causes the sexual dimorphism of autism. We employed a mass spectrometry (MS)-based phosphoproteomic approach to quantitatively profile differences in phosphorylation between inducible AC3 knockout (KO) and wild type (WT), male and female mice. In total, we identified 4,655 phosphopeptides from 1,756 proteins, among which 565 phosphopeptides from 322 proteins were repetitively detected in all samples. Over 46% phosphopeptides were identified in at least three out of eight biological replicas. Comparison of AC3 KO and WT datasets revealed that phosphopeptides with motifs matching proline-directed kinases' recognition sites had a lower abundance in the KO dataset than in WTs. We detected 14 phosphopeptides restricted to WT dataset (i.e., *Rabl6, Spast and Ppp1r14a*) and 35 exclusively in KOs (i.e., *Sptan1, Arhgap20, Arhgap44, and Pde1b*). Moreover, 95 phosphopeptides (out of 90 proteins) were identified only in female dataset and 26 only in males. Label-free MS spectrum quantification using Skyline further identified phosphopeptides that had higher abundance in each sample group. In total, 204 proteins had sex-biased phosphorylation and 167 of them had increased expression in females relative to males. Interestingly, among the 204 gender-biased phosphoproteins, 31% were found to be associated with autism, including *Dlg1, Dlgap2, Syn1, Syngap1, Ctnna1, Ctnnd1, Ctnnd2, Pkp4, and Arvcf*. Therefore, this study also provides the first phosphoproteomics evidence suggesting that gender-biased post-translational phosphorylation may be implicated in the sexual dimorphism of autism.

## Introduction

Primary cilia are tiny microtubule-based, membrane-ensheathed signaling devices present in most mammalian cells (Singla and Reiter, 2006). They depend on a special intraflagellar transport system for trafficking select cargo into and out of the cilium (Rosenbaum and Witman, 2002). Primary cilia are present in virtually every neuron in the brain, although they do not harbor synaptic junctions. Thus far, no ionotropic GABA_A_ receptor or glutamate receptors have been identified in neuronal cilia (Qiu et al., 2016; Sterpka and Chen, 2018). Although their physiological function is not well-understood (Louvi and Grove, 2011), defects in neuronal primary cilia lead to obesity, psychiatric diseases, intellectual disability, and neurodevelopmental disorders in humans (Fliegauf et al., 2007). Additionally, neuronal primary cilia have abundant expression of G-protein coupled receptors (GPCRs), such as type 3 somatostatin receptor (Wang et al., 2009; Einstein et al., 2010), type 6 serotonin receptor (Brodsky et al., 2017), and melanin-concentrating hormone receptor 1 (Green et al., 2012). This suggests that neuronal primary cilia depend on metabotropic signal pathways, rather than electrical input from synapses, to modulate neuronal activity. Most, if not all, ciliary GPCRs (Schou et al., 2015) are found to be either Gα_s_- or Gα_i_-protein coupled receptors (Qiu et al., 2016; Sterpka and Chen, 2018), which rely on heterotrimeric G-proteins, adenylyl cyclases, and cyclic adenosine monophosphate (cAMP) to send signals to the soma of neurons (Qiu et al., 2016; Sterpka and Chen, 2018).

AC3 represents a key enzyme mediating the cAMP signaling pathway in neuronal cilia (Bishop et al., 2007; Qiu et al., 2016) and is highly expressed in olfactory sensory cilia and in neuronal primary cilia throughout the central nervous system (CNS). It is known that AC3 in olfactory sensory neurons is essential for olfactory signal transduction in the main olfactory epithelium, and loss of AC3 leads to anosmia (loss of smell) (Wong et al., 2000). In the CNS, the physiological function of AC3 is yet to be established, but multiple lines of genetic evidence have associated AC3 with major depressive disorder (MDD) (Wray et al., 2012), obesity (Nordman et al., 2008; Stergiakouli et al., 2014), and autism spectrum disorders (ASD) (Skafidas et al., 2014; Yuen et al., 2017) in humans. Moreover, our previous studies have demonstrated that AC3 ablation in mice leads to pleiotropic phenotypes, including olfactory deficit (Wong et al., 2000; Chen et al., 2012), social interaction deficit (Chen et al., 2016), and depression-like behaviors (Chen et al., 2016). However, thus far it is unknown how AC3 or cAMP generated in neuronal primary cilia regulates signal transduction of central neurons.

Post-translational modifications (PTM) regulate signaling pathway and cellular processes, mediating intracellular communication and neuronal function. Protein phosphorylation is a major type of PTM, which can cause allosteric structure changes of proteins, activation, or inhibition of enzymes, alterations in protein's subcellular localization, and protein-protein interactions (Johnson, 2009). The major downstream effector protein of cAMP in cells is protein kinase A (PKA), whose activation leads to the phosphorylation of various proteins to propagate the cAMP signaling. We hypothesized that cAMP generated by AC3 locally in neuronal primary cilia can trigger a series of phosphorylation events, thereby modulating the structure and function of many downstream proteins and consequently affecting neuronal function. Therefore, identification of protein phosphorylation affected by AC3 will help delineate AC3-signaling network in CNS neurons. To systematically identify phosphorylation that is modulated by AC3, we employed a mass spectrometry-based quantitative phosphoproteomic approach, a powerful method to elucidate many signal pathways including the cAMP signaling pathway (Gunaratne et al., 2010; Roux and Thibault, 2013; Humphrey et al., 2015), to conduct a comparative phosphoproteomic profiling analysis. In this study, using a high-performance liquid chromatography-tandem mass spectrometry technology (HPLC-MS/MS), we identified thousands of peptides from prefrontal cortical tissues, some of which are differentially phosphorylated in AC3 wild type (WT) and knockout (KO) samples.

To date, although phosphoproteomic profiling analyses have identified a high throughput of phosphorylation sites (p-sites) in a variety of tissues, mouse strains, and different brain regions (Huttlin et al., 2010), virtually no studies have specifically compared phosphoproteomic differences between male and female samples. However, many neurodevelopmental disorders or psychiatric diseases such as MDD, attention deficit and hyperactivity disorder (ADHD) and ASD show a profound sex-bias (Halladay et al., 2015). For example, females have a higher risk of MDD than males (Labaka et al., 2018), whereas ASD affects more males than females with a male to female ratio of 4:1 (Kogan et al., 2009). It is unclear what causes the sexual dimorphism of these diseases. To evaluate the possibility that gender may differentially impact protein phosphorylation in the frontal cortex, we specifically compared phosphoproteomic datasets of two genders. This effort led to identification of over 200 proteins, whose phosphorylation were sex-biased. More female-biased phosphopeptides were identified than male-biased. Surprisingly, a high percentage of these targets (31%) are autism-associated proteins/genes, which include *Dlg1, Dlgap2, Syn1, SynGap1, Ctnna1*, and four delta catenin proteins (*Ctnnd1, Ctnnd2, Pkp4, and Arvcf*) (Yuan and Arikkath, 2017). Hence, this study provides the first phosphoproteomic evidence suggesting that gender-biased protein phosphorylation may contribute to the sexual dimorphism of autism.

## Materials and Methods

Supplemental Information provides additional Methods and Materials including Mice, Immunofluorescence Staining and Confocal Imaging, Western blot methods and detailed statistical methods.

### Tissue Preparation, Protein Extraction, and Phosphopeptide Enrichment

Prefrontal cortex tissues were isolated from 18 to 20 week old mice after euthanization, flash-frozen in liquid nitrogen, and stored in −80°C until analysis. Samples were then homogenized and lysed by grinding on ice in tissue lysis buffer (50 mM Tris-HCl, pH 8.0, 150 mM KCl, 1% TritonX-100, 0.5 mM PMSF, 0.5 mM EDTA) containing proteinase inhibitor cocktail (Cat. No. 04693159001, Roche, Germany) and phosphatase inhibitor cocktail (Cat. No. 04906837001, Roche, Germany). Lysates were cleared twice by centrifugation at 14K RPM for 20 min at 4°C. Protein centration was measured with Qubit fluorometer and ~3 mg of brain lysate from each sample was loaded in 12–20% gradient SDS-PAGE. In-gel digestion with reduction (final concentration 10 mM dithiothreitol, 56°C, 1 h) and alkylation (final concentration 55 mM iodoacetamide, 45 min in dark) were carried out at 37°C for 4 h. Phosphopeptides were enriched by MOAC (Titansphere^TM^ Phos-TiO Kit; GL Sciences Inc., Tokyo, Japan). Briefly, 500 μl of Speed Vac enriched trypsin digested peptides (1 mg/ml) were mixed with 1,000 μl binding solution (25% lactose acid, 60% acetonitrile, 0.3% trifluoroacetic), loaded onto Phos-TiO tip with 3 mg titanium dioxide (TiO_2_) resin. The resin was washed with 80% acetonitrile and 0.4% trifluoroacetic and eluted with 50 μl 5% ammonium hydroxide followed by 50 μl 5% pyrrolidine. Enriched phosphopeptides were concentrated via Speed Vac for pyrrolidine removal and mass spectrometric analysis.

### Mass Spectrometry and Database Searching

HPLC-MS/MS data was acquired on a LTQ Orbitrap Elite mass spectrometer (Thermo Fisher, CA) coupled to a NanoAccuity UPLC (Waters, MA) in Whitehead MS Facility at MIT (Boston, MA). Peptides were separated by a C18 column at 250 nL/min flow rate and 90-min gradient program. LC-MS data were acquired in an information-dependent acquisition mode, cycling between a MS scan (m/z 395–1,800, resolution 240,000) acquired in the Orbitrap, followed by 10 low-energy CID analysis in the linear ion trap. The centroided peak lists of the CID spectra were generated by PAVA (Guan and Burlingame, 2010) and searched against SwissProt.2017.11.01 *Mus Musculus* protein database, using Batch-Tag, a program module in Protein Prospector version 5.21.2 (University of California, San Francisco). A precursor mass tolerance of 20 ppm and a fragment mass tolerance of 0.6 Da were used for protein database search with S/T/Y phosphorylation included in variable modifications. Protein hits are reported with a Protein Prospector protein score ≥22, protein discriminant score ≥0.0 and a peptide expectation value ≤0.01 (Chalkley et al., 2005). With similar parameters, false discovery rate (FDR) of all samples was <1.5% when searched against the SwissProt random concatenated database. A threshold of SILP score > 6 was imposed for false phosphorylation site assignment <5%.

### Label-Free Quantification

Label-free quantification was performed using Skyline ver 4.1.0.18169 via MS1 full-scan filtering with the library generated by ProteinProspector (Cut-off score = 0.95; Precursor charge = 2, 3, 4, 5; Max Miss Cleavages = 1) and the SwissProt Mus Musculus protein FASTA file (Schilling et al., 2012). MS results of three fractions from each sample were combined into one project. Peak areas of identified peptides were generated from Skyline and normalized to the protein concentration of lysate samples. Phosphopeptides with different phosphorylation states, such as mono-phosphorylation and di-phosphorylation, were considered as different entries for quantitation. Identical phosphopeptides from different gel fractions of a same sample were combined for quantitation. Since methionine oxidation can be introduced during sample handling, phosphopeptides with different methionine oxidation states were combined for quantitation. Phosphopeptides with identical sequence in homologous proteins were included in the calculation of protein phosphorylation level for homologous proteins.

### Bioinformatics Analysis

The phosphoprotein lists generated from ProteinProspector were analyzed by AmiGO 2 (Mi et al., 2017) for pathway/network enrichment. The kinase substrate motif search was performed by web-based Motif-X v1.2 10.05.06 (motif-x.med.harvard.edu/motif-x.html) and analyzed basing on the Human Protein Reference Database (www.hprd.org) (Keshava Prasad et al., 2009; Chou and Schwartz, 2011). Phosphopeptides with site assignment confidence level higher than 95% were aligned in Motif-X. The motif widths were adjusted to 6 amino acids from each side of the phosphorylation site. The occurrences were set as 5 and significances were set as 0.000004, which led to a maximal number of motifs and *p* < 0.001. Protein-protein interaction network analysis was performed by the Cytoscape-based Search Tool for the Retrieval of Interacting Genes/Proteins (STRING, string-db.org) (Szklarczyk et al., 2015). All the proteins with phosphorylation that revealed differences between AC3 KO and WT, or between female and male were searched in PubMed and AutDB (Autism Gene Database, updated in Sept. 2018) (Basu et al., 2009), an autism candidate gene database, to explore possible association between the disease and phosphoproteome.

### Data Analysis

Data analysis and figure constructs were performed with Origin Pro and Graphpad Prism 7 software for Student's *t*-test, normality test. Phosphopeptides detected in no <3 KO samples but none in any WT control samples (out of *n* = 8 pair) were considered statistically significantly enriched in AC3 KO sample group (determined by Two Population Proportions Comparison). For phosphopeptides that were detected in both genotypes or genders, label-free quantitation of was used to identify statistically significant (*p* < 0.05) differences in phosphorylation between KO and WT, or female and male. Phospho-peptides with $\ln\;\frac{peak\;area\;of\;KO\#}{average\;peak\;area\;of\;all}\leq 0$ showed lower phosphorylation levels of the target peptide site in AC3 KO samples than in WT samples. Conversely, $\ln\;\frac{peak\;area\;of\;KO\#}{average\;peak\;area\;of\;all}\geq \;0$ showed higher phosphorylation levels of the target phosphopeptide site in AC3 KO samples than in WT samples. For label-free quantification between two genders, phosphopeptides detected in 3 of 8 gender pairs covered both of two genders were analyzed. Phosphopeptides with $\ln\;\frac{\;peak\;area\;of\;F\#}{average\;peak\;area\;of\;all}\leq 0$ and $-\ln\;\frac{\;peak\;area\;of\;M\#}{average\;peak\;area\;of\;all}\leq 0$ had lower phosphorylation levels in female samples than in male samples. In vice versa, $\ln\;\frac{\;peak\;area\;of\;F\#}{average\;peak\;area\;of\;all}\geq 0$ and $-\ln\;\frac{\;peak\;area\;of\;M\#}{average\;peak\;area\;of\;all}\geq 0$ means phosphorylation levels of target phosphopeptide site in female samples were higher than that in males. All peptides spectra presented in the figure and table were reviewed and verified manually. If not otherwise indicated in the figure legends, statistical analysis was a paired student *t*-test with a two-tailed distribution. n.s. not significant, ^*^p < 0.05, ^**^p < 0.01, ^***^ *p* < 0.001. Data were considered as statistically significant if *p* < 0.05. Data in the graph were presented as mean ± standard error of the mean.

### Two Population Proportions Comparison

We used “Two Population Proportions” for comparison to set the “3 out of the *n* = 8 samples” cut-off to determine “a phosphopeptide is enriched in one sample group.” Mass spectrometer randomly picks a peptide separated by HPLC to be sequenced and identified. High abundance peptides are detected more frequently. We calculated the *p*-value by comparing two population proportions between two groups as the following. Specifically, we wanted to test the null hypothesis of *p*_1_ = *p*_2_ against the research hypothesis of *p*_1_≠*p*_2_ in the following.

For an individual phosphopeptide, in control group, $\hat{p_{1}}=\frac{X_{1}}{n_{1}}=\frac{3}{8}$; in AC3 KO group, $\hat{p_{2}}=\frac{X_{2}}{n_{2}}=\frac{0}{8}$; in the combined group $\hat{p}=\frac{X_{1}+X_{2}}{n_{1}+n_{2}}=\frac{3}{16}$; We calculated the test statistics value according to the z-score $z=\frac{\hat{p_{1}}-\hat{p_{2}}}{\sqrt{\hat{p}\left(1-\hat{p}\right)\left(\frac{1}{n_{1}}+\frac{1}{n_{2}}\right)}}=1.92$, and used the standard normal distribution, N (0,1) to approximate the *p* = *P* (N (0, 1) > *z*) = 0.027 < 0.05. Similarly, if a phosphopeptide was detected in 2 control samples and was not detected in any KO samples, then z-score = 1.52, *p* = 0.0655. If a phosphopeptide was detected in three control samples and detected in 1 KO sample, *Z* = 1.62, *p* = 0.0526. Conclusion: If a phosphopeptide was detected in three or more than three control samples (out of *n* = 8), but in none of 8 KO samples, then the proportion that this phosphopeptide in the Control group was significantly higher than that in the KO group. Similarly, this calculating method was applied to the other three groups (KOs, females, and males).

### Statistics to Determine “More Female-Biased Phosphorylation Than Male-Biased”

We used two statistical methods “Two Population Proportions” comparison and “Student *t*-test” to determine if there were “more female-biased phosphorylation than male-biased.” Using Two Population Proportions comparison, we calculated a z-score = 9.87, *p* < 0.0001. Conducting an unpaired Student *t*-test yielded a *p* = 0.023 < 0.05. Detailed methods were provided in the Supplemental Information.

### Statistics to Determine “A High Percentage of the Sex-Biased Phosphoproteins Are From ASD-Associated Genes”

AutDB collects a total of 1,053 ASD gene entries. The human genome is estimated to have 20,000 genes. Thus, the 1,053 ASD genes are estimated to represent 5.2% of all human genes in human genome. We have identified 204 sex-biased phosphorylation, among which 32 proteins (15.6%) were listed in the AutDB as ASD genes. The ASD gene percentage is 32/204 = 15.6%. We used “Two Population Proportions” method to compare two groups and produced a *Z*-score = 6.57 and *p* < 0.0001. Detailed statistical method was provided in the Supplemental Information.

### Statistics of Cross Comparison of Four Groups

We used Pearson's Chi-square test to carry out a cross comparison of four groups (Female KOs, Females WTs, Male KOs, Male WTs). Detailed method as well as the results of cross comparison of 4 groups were provided in the Supplemental Information.

### Choosing Appropriate Statistical Method for Data Comparison

Throughout this manuscript, we mostly used two population proportions comparison (3 out of *n* = 8 cut off) as well as Student's *t*-test for data comparison. Rationales were provided in the Supplemental Information.

## Results

### AC3 Is Highly Enriched in Neuronal Primary Cilia, but Not in Astrocyte Cilia or Microglia in the Prefrontal Cortex

**Figure 1:**
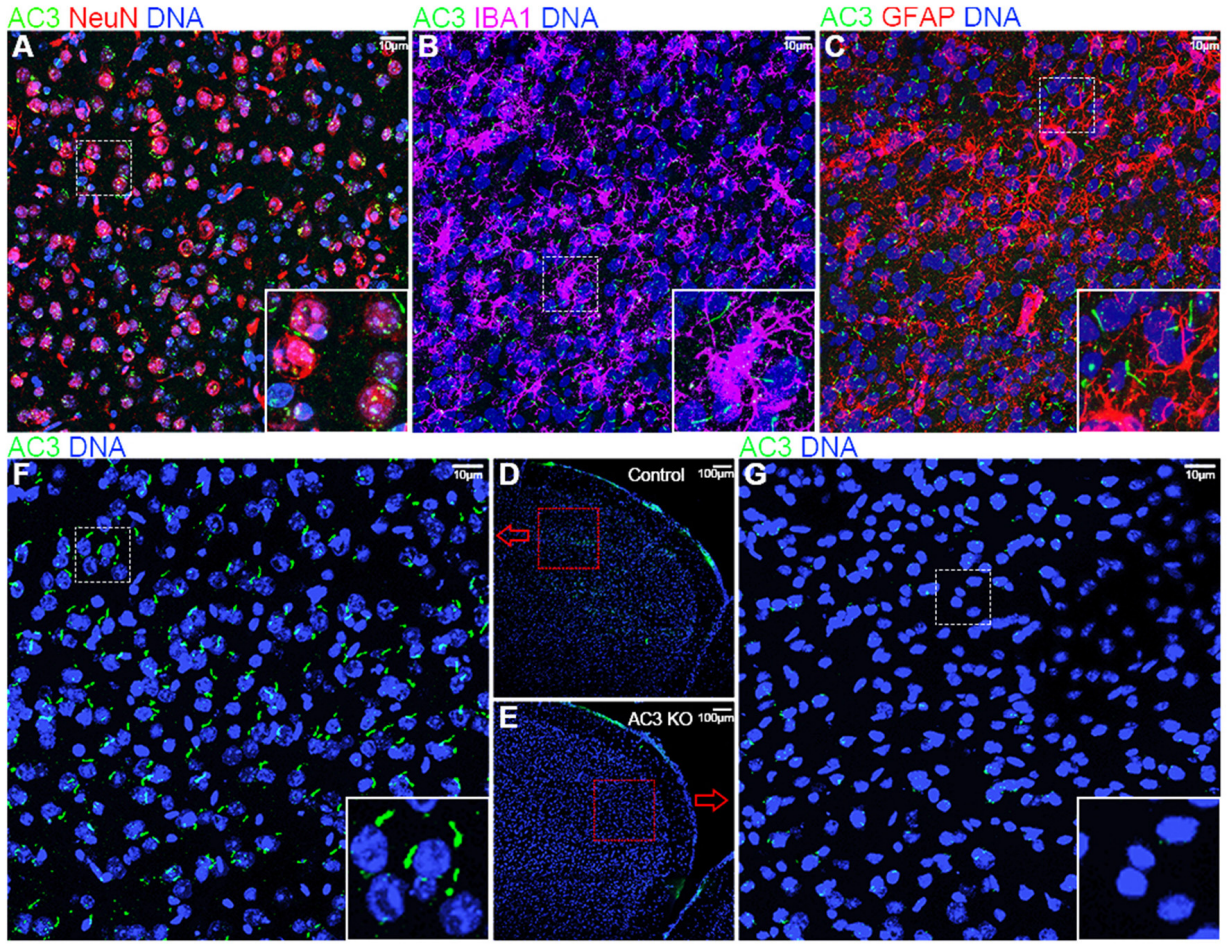
Immunofluorescence staining of AC3 in the mouse prefrontal cortex. AC3 was predominantly enriched in neuronal primary cilia in the prefrontal cortex, but not microglia or astrocyte cilia. **(A)** Co-staining of NeuN (a neuronal marker, red) and AC3 (green) demonstrates that AC3 in primary cilia mostly localize very closely with neuronal soma (red). **(B)** Co-staining of IBA1 (a microglia marker, magenta) with AC3 (green) demonstrates the absence of AC3 in microglia. Note that green staining generally does not overlap with magenta. **(C)** Co-staining of GFAP (an astrocyte marker, red) with AC3 (green) shows that most astrocytes do not have AC3 in cilia. **(D–G)** AC3 was highly expressed neuronal primary cilia in AC3 WT **(D,F)**, but minimally in AC3 inducible KO tissues **(E,G)**. **(F,G)** are enlarged from **(D,E)**, respectively.

Because AC3 is associated with MDD (Wray et al., 2012; Chen et al., 2016) and ASD (Skafidas et al., 2014; Yuen et al., 2017), we chose the prefrontal cortex, a brain region important for social behaviors, personality and emotion, and mood state (Siddiqui et al., 2008), in our phosphoproteomics analysis. First, we tested if AC3 is expressed in the prefrontal cortex and if so, in which cell types it is expressed. Immunofluorescence staining images demonstrate that AC3 is highly expressed in neuronal primary cilia (Figure 1A), but not microglia (Figure 1B) or astrocytic primary cilia (Figure 1C) in the prefrontal cortex. These results are consistent with previous reports (Kasahara et al., 2014; Sipos et al., 2018; Sterpka and Chen, 2018), showing that AC3 is primarily confined to neuronal primary cilia in the brain. These data also suggest that AC3 mostly modulates the function of neurons, but not of astrocytes or microglia.

Given the major target protein of cAMP in cells is PKA, which can phosphorylate numerous downstream proteins, we set out to determine if AC3 affects post-translational phosphorylation in neurons in the prefrontal cortex. To circumvent developmental complications, we utilized AC3 floxed:Ubc-Cre/ERT2 KO mouse strain (Chen et al., 2016) to ablate AC3 temporally in adult mice. Ubc-Cre/ERT2 is a tamoxifen-inducible Cre strain with Cre expression driven by the ubiquitin C promoter (Ruzankina et al., 2007). Administration of tamoxifen to the AC3 floxed:Ubc-Cre/ERT2 mice removed more than 95% AC3's immunostaining signal in the prefrontal cortex (Figure 1F), whereas vehicle injection had no effect (Figure 1G), demonstrating that AC3 was successfully ablated in the adult mouse brain after tamoxifen injection.

### Mass Spectrometry-Based Phosphoproteomic Analysis Using AC3 WT and KO, Male and Female Samples

**Figure 2:**
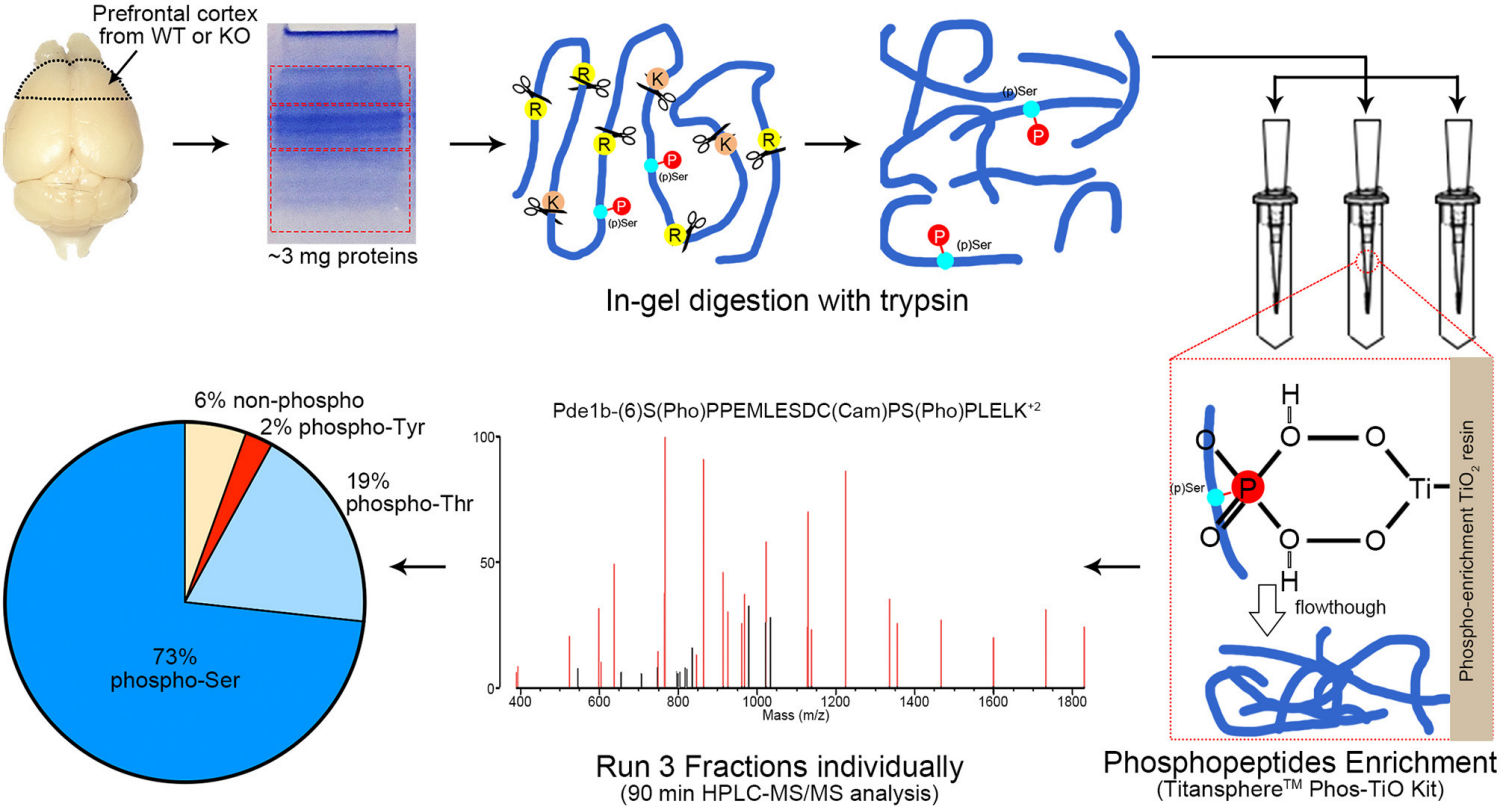
Workflow of phosphoproteomics analysis. Prefrontal cortex tissue homogenizations were separated via SDS-PAGE and cut into 3 fractions with equal protein amount (according to Coomassie blue staining intensity measured by ImageJ). Protein extraction via in-gel digestion with trypsin (1:300 enzyme/substrate) was performed on ~3 mg protein for each sample. Phosphopeptides from each fraction were enriched by Titansphere^TM^ Phos-TiO_2_ Kit and analyzed with HPLC-MS/MS for 90 min, respectively. A MS2 spectra of Pde1b (p)Ser7 (p)Ser18 is shown as an example. MS spectra data were analyzed by ProteinProspector (ver. 5.21.2, UCSF, San Francisco, CA) and Skyline (ver. 4.1.0.18169). Among all MS-detected phosphopeptides (94%), 73% are (p)Ser, 19% (p)Thr, and 2% (p)Tyr. *n* = 16, 4 female WTs, 4 female KOs, 4 male WTs, and 4 male KOs.

To efficiently identify phosphorylation differences in the prefrontal cortex between AC3 KO and WT mice, we utilized a MS-based phosphoproteomic approach to identify phosphopeptides in large scale. Proteins of the prefrontal cortex (isolated from WT, KO, male, and female mice, respectively) were extracted and digested with trypsin. Resultant phosphopeptides were enriched using TiO_2_ enrichment column and then subjected to HPLC-MS/MS analysis and database search for identification using UCSF Protein Prospector (Figure 2). Additionally, we determined the relative abundance of (p)Ser, (p)Thr, and (p)Tyr residues in the dataset. On average, ~94% of detected unique peptides were phosphorylated peptides, indicating that the phosphopeptide TiO_2_ enrichment was efficient and successful (Figure 2). Among all phosphorylated peptides (total 94%), (p)Ser accounted for 73% of total phospho-peptides, (p)Thr 19%, and (p)Tyr 2% (Figure 2), which is consistent with previous phosphoproteomic reporting (Olsen et al., 2006; Huttlin et al., 2010).

**Figure 3:**
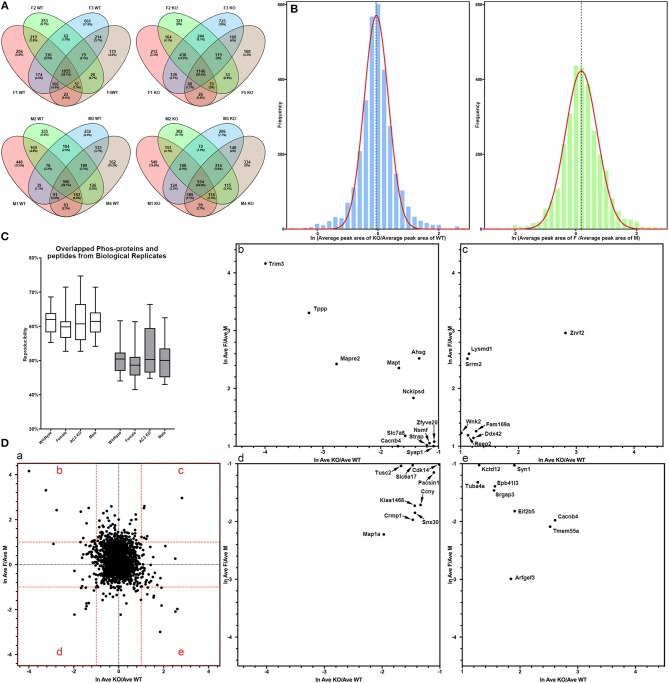
Data quality of phosphoproteomic analysis. **(A)** Venn-diagram of phosphorylated peptides detected from 4 groups (female KO, female WT, male KO, male WT). In each group, around 62% of phosphorylated peptides were detected in more than 2 of 4 samples, and around 27% of phosphorylated peptides were detected in all 4 samples. **(B)** Histogram of relative phosphopeptide abundance of KOs compared to WTs (left), and females compared to males (Autism Genome Project et al., 2007). The histogram indicates that the dataset fits into a normal distribution. The X-axis denotes average ratio of phosphopeptide intensity in AC3 KO relative to WT (left), or females relative to males (Autism Genome Project et al., 2007). **(C)** Peptide and protein repeatability. The 28 random pairs of our biological replicates (8 per groups) showed a range for peptide-level repeatability as 43–62% and a range for protein-level repeatability as 53–75%. **(D)** Scatter plot of phosphopeptide abundance of KOs relative to WTs, and of females relative to males. (**D-**a) The X-axis denotes average ratio of phosphopeptide intensity of AC3 KOs relative to WTs. The Y-axis denotes average ratio of phosphopeptide intensity of females relative to males. (b–e) Zoom-in plot of (a) for phosphopeptides that are highly enriched in female WTs (b); female KOs (c); male WTs (d); and male KOs (e). Cut-off lines ln (KO/WT) or ln (female/male) were set to 1, meaning over 2.7-fold increase in phosphopeptide MS1 spectra intensity. The majority of p-sites exhibit similar expression levels, whereas some p-sites have different phosphorylation levels among AC3 KOs and WTs, or males and females.

In total, 4,655 different phosphopeptides were detected from 1,756 proteins (Table S1), among which 2,390 (51.4%) were present at least in three out of the 8 biological replicas in AC3 KO group, 2,244 (48.2%) in AC3 WT group, 2,427 (52.2%) in Female group, 2,158 (46.4%) in Male group (Table S2). Five hundred and sixty five phosphorylation sites from 322 proteins were detected out of all 16 samples (Table S3). In each sample group (*n* = 4 for each), 62% of phosphorylated peptides were detected in more than 2 of 4 samples, and 27% of phosphorylated peptides were detected in all 4 samples (Figure 3A), demonstrating reproducibility of the phosphoproteomic analysis. Note that the same peptide having two p-sites was counted as two different modifications. Histograms of the average of eight pairs of KO/WT MS1 peak area and 8 pairs of female/male MS1 peak area demonstrate that our datasets largely fell in approximate statistic normal distribution (Figure 3B). Our experiments using biological replicas generated a range of 43–62% for peptide-level repeatability and a range of 53–75% for protein-level repeatability, representing a good range for phosphoproteomics analyses (Figure 3C). Forty-six to fifty-two percentages of phosphopeptides were present at least in three out of the 8 biological replicas in one sample group. These numbers were consistent with previous report (35–60%) and verified data solidity (Tabb et al., 2010).

For global phosphoproteomic profiling, the average MS1 peak area of KOs relative to WTs (x-axis), and the average MS1 peak area of males relative to females (y-axis) were constructed into a scatter plot (Figure 3D). This shows that the phosphorylation levels of most peptides had no general difference between genotypes or between genders, whereas only some modifications exhibit marked differences between AC3 KOs and WTs, or between females and males (Figure 3D). To validate our phosphoproteomic data, we also looked into Western blotting as an orthogonal method, but we were limited by the availability of commercial antibodies. We chose three anti-phosphopeptide antibodies (anti-pCaMK2a/b T286/287, anti-pSyn1 S605, anti-pERK1/2 T203/Y205 T183/Y185) for Western blot assays to verified their MS data. The anti-pCaMK2 antibody recognizes both the CaMK2a (p)Thr 286. The Western blot signal shows that CaMK2a (p)Thr 286 had no difference between WT and KO samples, and between male and female samples, which were consistent with their MS1 quantification data (Figure S1). Two other antibodies (anti-pSyn1 and anti-pERK1/2) yielded similar results (Figure S1).

To determine which classes of proteins were enriched in our MS-based phosphoproteomic analysis, the dataset of all phosphopeptides was subjected to Gene Ontology (GO) enrichment analysis (Mi et al., 2017). The GO analysis demonstrated that synaptic vehicle exocytosis proteins, gluconeogenesis, synaptic membrane protein, SNARE complex, tubulin, kinases, and SNAP receptor activity were highly enriched in our dataset, while G protein-coupled receptors, protease inhibitors, extracellular space proteins, ligand-gated ion channels, proteins mediating immune responses, and phagocytosis, cell recognition proteins, transferase, and nuclease activity were under-represented (Figure S2).

### Motifs Matching Proline-Directed Kinases' Substrate Motifs Had Decreased Abundance AC3 KO Samples

**Figure 4:**
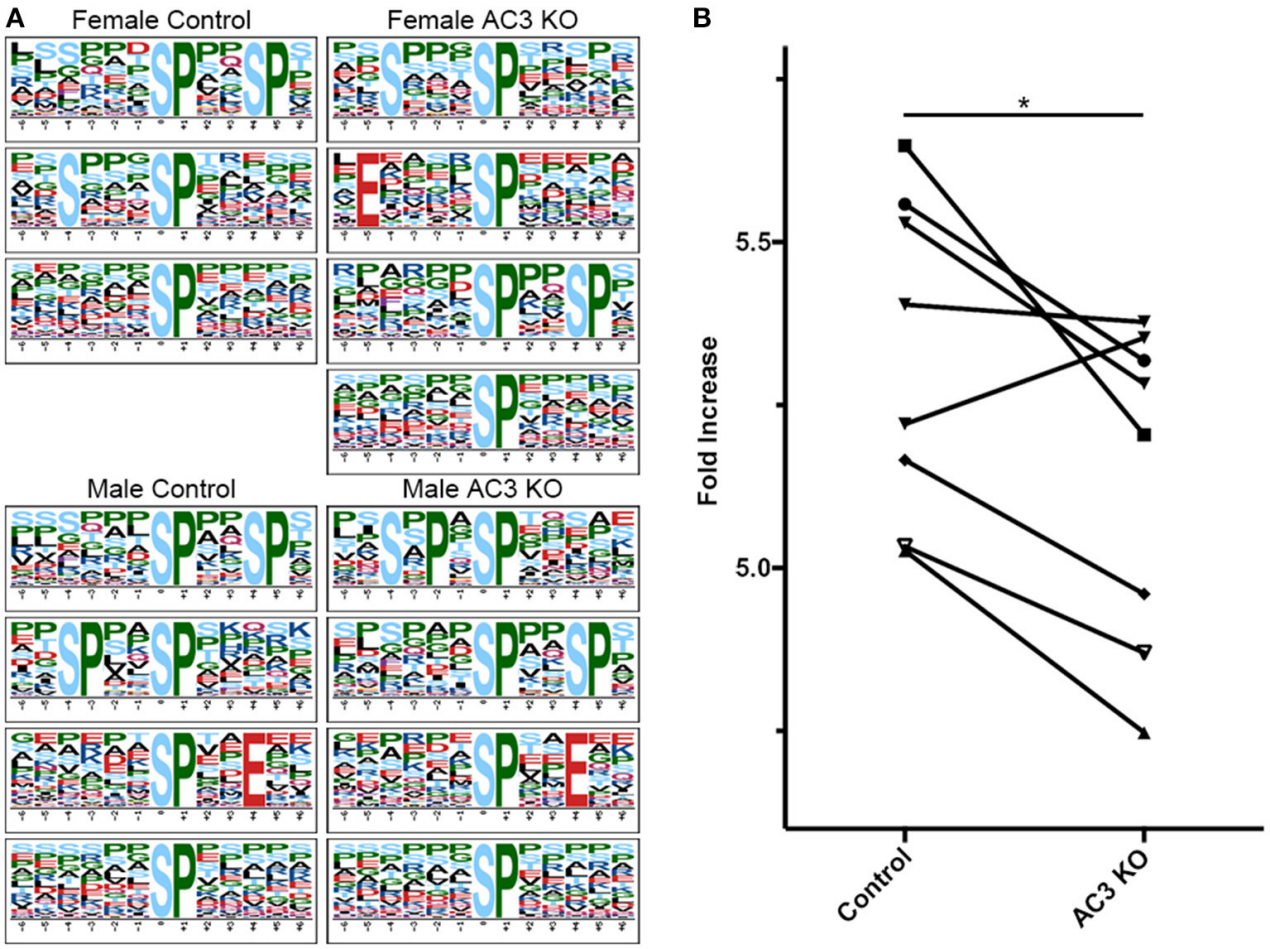
Proline-directed kinase recognized peptide abundance is decreased in AC3 KOs compared to WTs. The sequence logos for (p)Ser with a proline residue at the +1 position for 4 different sample groups. **(A,B)** comparison of proline-detected specific motifs in enrichment fold between AC3 KOs and WTs (*n* = 8 pairs, 4 male pairs and 4 female pairs, ^*^P < 0.05).

cAMP regulates many kinases' activity including PKA and ERK1/2 (Waltereit and Weller, 2003; Sassone-Corsi, 2012). To classify MS-identified phosphopeptides into different motif categories and to determine if AC3 ablation affects the overall activity of certain group of kinases, we used Motif-X (see Figure S3 for detailed method workflow) to determine the abundance of phospho-motifs in our samples, which infers corresponding kinases' activity (Pinna and Ruzzene, 1996). Motif-X is a software tool to extract overrepresented motifs from all phosphopeptide sequences of a dataset (Chou and Schwartz, 2011). Out of the four sample groups, we found that proline-directed kinase's substrate motifs were the most common motifs in our samples (37% of all p-sites in AC3 KOs and 38% of all p-sites in WTs), largely in line with previous report (Huttlin et al., 2010). The motif […(p)S-P…] was highly enriched in all four groups of samples (Figure 4A). Moreover, we compared the Motif-X sequence logos with Mouse International Protein Index (IPI) database and found that motifs ([Sxxx(pS)P], [Exxxx(pS)P], [(pS)xxSP], [SxPx(pS)P], [(pS)PxxE], [(pS)P]) were enriched in AC3 KO mice, while motifs ([Sxxx(pS)P], [(pS)xxSP], [(pS)P], [SPxx(pS)P], [(pS)PxxE]) were more abundantly present in AC3 WT mice (Figure 4A). Notably, the enriched proline-directed kinase-recognized motifs in our dataset matched to the kinase substrate motifs of GSK-3, CDK5, ERK1, ERK2, and mitogen-activated protein kinase-activated protein kinase 3 (Mapkapk3) reported on the Human Protein Reference Database (http://hprd.org/serine_motifs). We discovered that the motifs matching proline-detected kinases' recognition sites had significantly higher abundance in AC3 WT than in AC3 KO mice (Figure 4B), and the difference was gender unspecific. These data suggest that ablation of AC3 decreases the overall activity of proline-directed kinases in the prefrontal cortex.

### Differential Expression of Phosphopeptides in AC3 KO and WT Mice

**Figure 5:**
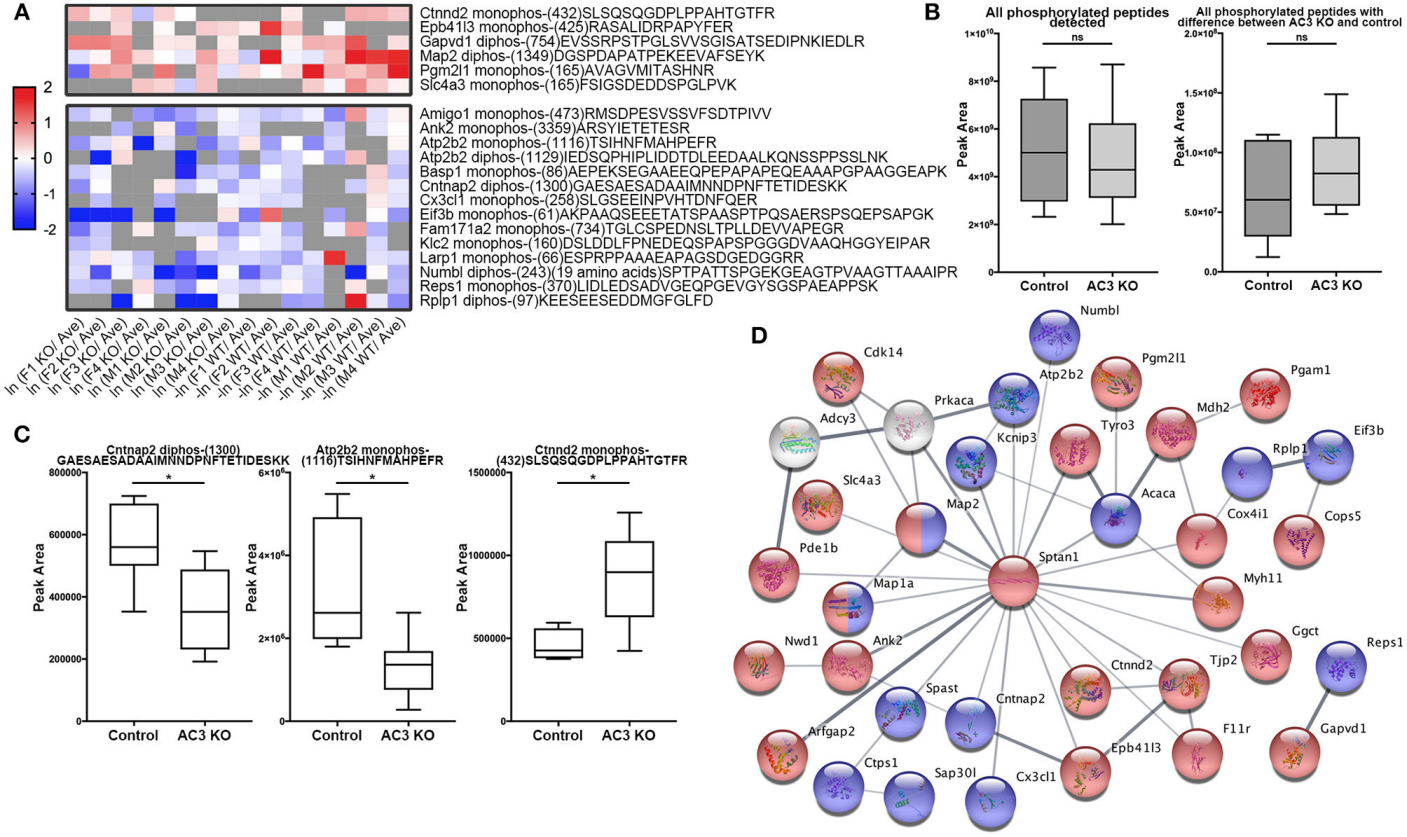
Comparison of phosphopeptide abundance in AC3 KO and WT datasets and their protein interaction. **(A)** Heat map of phosphopeptides that show significant differences in AC3 KOs or WTs. X-axis denotes ln (peak area of individual peptide from KO data/average peak area of all data) for AC3 knockout dataset, or -ln (peak area of individual peptide from WT data/average peak area of all data) for AC3 WT dataset. Peak area differences of phosphopeptides in KO and WT datasets with p < 0.05 (unpaired Student's *t*-test) was used to construct the heat map. **(B)** The sum peak areas of all identified phosphopeptides in AC3 WTs (*n* = 8) and AC3 KOs (*n* = 8) have no difference (left). The sum peak areas of 20 phosphopeptides (shown in **A**) in AC3 controls and AC3 KOs have no significant difference (unpaired Student's *t*-test). **(C)** Three representative plots phosphopeptides from *Cntnap2, Atp2b2*, and *Ctnnd2* of label-free quantification of phosphorylation levels in WTs and KOs. **(D)** Protein-protein interaction STRING analysis using phosphoproteins differentially expressed in AC3 KOs and WTs. AC3 and PKA were manually included. Phosphoproteins with increased phosphorylated levels in KOs were highlighted in red, or in WTs highlighted in blue. Proteins marked in half red/blue had both increased and decreased p-sites on different positions in both WTs and KOs. Interaction score confidence = 0.400.

To identify phosphopeptides that were differently expressed in AC3 KO and WT mice, we compared AC3 KO and WT datasets. Phosphopeptides that were expressed differently (*p* < 0.05 by Two Populations Proportions comparison) in AC3 WT and KOs were pooled in Tables 1, 2. Table 1 lists 14 phosphopeptides (from 14 proteins/genes such as *Map2* and *Spast*) that were identified only in AC3 WT dataset. Table 2 contains 35 phosphorylation sites (out of 35 genes including *Arhgap20, Arhgap44, Dlgap2, Pde1b, and Sptan1*) that were exclusively detected in AC3 KO dataset. In addition to the presence-or-absence detection, phosphopeptides' mass spectra in a dataset also exhibit variant abundance in different samples, reflecting the phosphopeptides expression levels in tissues. To determine if there are quantitative spectra differences between AC3 WT and KO mice, we used Skyline software (Schilling et al., 2012) to conduct label-free quantification. The Skyline-guided quantification was based on the MS1 peptide level. After completion of the MS1 qualification, we used differentially expressed peptides in WTs and KOs to construct a heat map. Comparison of WT data with KO data led to identification of 20 phosphopeptides (from 19 proteins/genes), which had significantly different phosphorylation levels (*p* < 0.05 by unpaired Student's *t*-test) between AC3 WT and KO dataset (Figure 5A). Six of them had higher expression in AC3 KOs (Figure 5A, Top), whereas 14 of them (from 13 proteins/genes) were more abundantly expressed in WTs (Figure 5A, Bottom). The overall phosphorylation levels of all phosphopeptides and the 20 differentially expressed phosphopeptides have no significant difference between AC3 WT and KO datasets (Figure 5B). Figure 5C shows that three representative phosphopeptides (identified from *Cntnap2, Atp2b2*, and *Ctnnd2*) exhibited significant different MS1 peak area between WT and KO dataset.

**Table 1:** Phosphopeptides exclusively detected in AC3 WT dataset.

**Gene**	**Protein name**	**Detection times (WT)**	**Peptide**	**References for ASD or related disorders**
Acaca^*^	Acetyl-CoA carboxylase 1	3	monophos-(18)FIIGSVSEDNSEDEISNLVK	Girirajan et al., 2013
Map1a^*^	Microtubule-associated protein 1A	4	monophos-(2586)AKPASPARR	Myers et al., 2011
Map2	Microtubule-associated protein 2	3	monophos-(1004)ELITTKDTSPEK	Mukaetova-Ladinska et al., 2004
Spast	Spastin	3	diphos-(89)SSGTAPAPASPSPPEPGPGGEAESVR	Talkowski et al., 2012
Ahsg	Alpha-2-HS-glycoprotein	3	monophos-(301)HAFSPVASVESASGETLHSPK	#N/A
Bloc1s3	Biogenesis of lysosome-related organelles complex 1 subunit 3	3	diphos-(51)VAGEAAETDSEPEPEPTVVPVDLPPLVVQR	#N/A
Ctps1	CTP synthase 1	3	diphos-(570)SGSSSPDSEITELKFPSISQD	#N/A
Kcnip3	Calsenilin	3	diphos-(49)WILSSAAPQGSDSSDSELELSTVR	#N/A
Ppp1r14a	Protein phosphatase 1 regulatory subunit 14A	4	monophos-(19)ARGPGGSPSGLQK	#N/A
Rabl6	Rab-like protein 6	4	monophos-(477)NISLSSEEEAEGLAGHPR	#N/A
Sap30l	Histone deacetylase complex subunit SAP30L	3	diphos-(89)KASDDGGDSPEHDADIPEVDLFQLQVNTLR	#N/A
Slc6a20b	Sodium- and chloride-dependent transporter XTRP3B	3	monophos-(0)MESPSAHAVSLPEDEELQPWGGAGGPGQHPGRPRSTECA HPGVVEK	#N/A
Ube2v1	Ubiquitin-conjugating enzyme E2 variant 1	3	monophos-(135)LPQPPEGQCYSN	#N/A
Znf281	Zinc finger protein 281	3	monophos-(0)MKIGSGFLSGGGGPSSSGGSGSGGSSGSASGGSGGGR	#N/A

Phosphopeptides listed in the table were detected in no <3 times in AC3 WT dataset (n = 8), but never detected in any AC3 KO dataset (n = 8). Proteins on top of the table were annotated in the AutDB (or per PubMed literature, labeled with

* *) to be associated with ASD or neurodevelopmental disorders. The number in brackets indicates the position of amino acid just before the peptide*.

**Table 2:** Phosphopeptides exclusively detected in AC3 KO dataset.

**Gene**	**Protein name**	**Detection times (KO)**	**Peptide**	**References for ASD or related disorders**
Dlgap2	Disks large-associated protein 2	3	monophos-(717)CSSIGVQDSEFPDHQPYPR	Girirajan et al., 2013
Hepacam	Hepatocyte cell adhesion molecule	5	monophos-(316)DKDSSEPDENPATEPR	Myers et al., 2011
Lsm14a^*^	Protein LSM14 homolog A	3	monophos-(214)RSPVPARPLPPTSQK	Mukaetova-Ladinska et al., 2004
Map1a^*^	Microtubule-associated protein 1A	3	monophos-(457)KFSKPDLKPFTPEVR	Talkowski et al., 2012
Map2	Microtubule-associated protein 2	4	monophos-(1634)SGILVPSEK	Girirajan et al., 2013
Myh11^*^	Myosin-11	4	monophos-(1946)VIENTDGSEEEMDAR	Myers et al., 2011
Nav1^*^	Neuron navigator 1	3	monophos-(374)LELVESLDSDEVDLK	Mukaetova-Ladinska et al., 2004
Sptan1^*^	Spectrin alpha chain, non-erythrocytic 1	4	diphos-(1181)DEADSKTASPWK	Talkowski et al., 2012
Strip2^*^	Striatin-interacting proteins 2	3	monophos-(361)QDSLDIYNER	Girirajan et al., 2013
Agk	Acylglycerol kinase, mitochondrial	3	monophos-(281)LASFWAQPQDASSR	#N/A
Arfgap2	ADP-ribosylation factor GTPase-activating protein 2	4	monophos-(428)AISSDMFFGR	#N/A
Arhgap20	Rho GTPase-activating protein 20	3	diphos-(795)SKPVPISVASYSHGSSQDHPRK	#N/A
Arhgap44	Rho GTPase-activating protein 44	3	monophos-(604)GSPGSIQGTPCPGTQLGPQPAASPSQLPADQSPHTLR	#N/A
C2cd2l	C2 domain-containing protein 2-like	3	monophos-(413)NLGTPTSSTPRPSITPTK	#N/A
Cdk14	Cyclin-dependent kinase 14	3	monophos-(92)VHSENNACINFK	#N/A
Clip2	CAP-Gly domain-containing linker protein 2	3	monophos-(914)VLLLEANRHSPGPER	#N/A
Cops5	COP9 signalosome complex subunit 5	3	monophos-(282)GSFMLGLETHDR	#N/A
Cox4i1	Cytochrome c oxidase subunit 4 isoform 1, mitochondrial	3	monophos-(42)DYPLPDVAHVTMLSASQK	#N/A
F11r	Junctional adhesion molecule A	3	diphos-(278)VIYSQPSTRSEGEFK	#N/A
Fam126a	Hyccin	3	monophos-(452)SFEQVSGAPVPR	#N/A
Ggct	Gamma-glutamylcyclotransferase	3	monophos-(169)GKISDEMEDIIK	#N/A
Itm2c	Integral membrane protein 2C	3	monophos-(20)AAASGPASASAPAAEILLTPAR	#N/A
Kcnb2	Potassium voltage-gated channel subfamily B member 2	3	monophos-(460)SMELIDVAVEK	#N/A
Kctd8	BTB/POZ domain-containing protein KCTD8	3	monophos-(410)RNSELFQSLISK	#N/A
Lysmd2	LysM and putative peptidoglycan-binding domain-containing protein 2	3	monophos-(28)SRSTSEPEEAELSLSLAR	#N/A
Mdh2	Malate dehydrogenase, mitochondrial	4	monophos-(241)AGAGSATLSMAYAGAR	#N/A
Nwd1	NACHT and WD repeat domain-containing protein 1	3	monophos-(935)LWSLLSGQEKVTILDGGSQNPTEPQSWDLHVDER	#N/A
Pde1b	Calcium/calmodulin-dependent 3',5'-cyclic nucleotide phosphodiesterase 1B	4	diphos-(6)SPPEMLESDCPSPLELK	#N/A
Pgam1	Phosphoglycerate mutase 1	3	monophos-(117)SYDVPPPPMEPDHPFYSNISK	#N/A
Pja1	E3 ubiquitin-protein ligase Praja-1	3	monophos-(226)VFFDTDDDDDVPHSTSR	#N/A
Serbp1	Plasminogen activator inhibitor 1 RNA-binding protein	3	monophos-(240)QISYNCSDLDQSNVTEETPEGEEHPVADTENKENEVEEVK	#N/A
Srcin1	SRC kinase signaling inhibitor 1	3	diphos-(1124)AVSEVVRPASTPPIMASAIKDEDDEER	#N/A
Stac2	SH3 and cysteine-rich domain-containing protein 2	3	monophos-(45)SKSVENFFLR	#N/A
Tjp2	Tight junction protein ZO-2	3	monophos-(965)DASPPPAFKPEPPK	#N/A
Tyro3	Tyrosine-protein kinase receptor TYRO3	3	monophos-(799)AEQPTESGSPEVHCGER	#N/A

Phosphopeptides listed in the table were detected in no <3 times in KO dataset (n = 8), but never detected in any AC3 WT dataset (n = 8). Proteins on top of the table were annotated in the AutDB (or per PubMed literature, labeled with

* *) to be associated with ASD or neurodevelopmental disorders. The number in brackets indicates the position of amino acid just before the peptide*.

In total, we identified 65 proteins either with phosphopeptides exclusively present in AC3 WTs or KOs (as listed in Tables 1, 2) or with phosphopeptide abundance having significant differences in WTs or KOs (as shown in Figure 5A). Since *ADCY3* is associated with MDD (Wray et al., 2012; Chen et al., 2016) and ASD (Skafidas et al., 2014; Yuen et al., 2017), we asked if AC3 affects phosphorylation of other autism-associated proteins. While MDD does not have a candidate gene databank thus far, ASD does have several databases, among which AutDB provides rich resources on ASD candidate genes (http://autism.mindspec.org/autdb/search) (Basu et al., 2009). We used these 65 proteins to search against AutDB as well as PubMed and found that 9 out of 65 were annotated in AutDB and other 7 were annotated in PubMed to be associated with ASD, which include phosphopeptides from *Dlgap2, Map2, Ank2, Ctnnb2, Cntnap2, Sptan1*, and *Spast* (Tables 1, 2 and Figure 5A). To explore if *Adcy3* ablation affects ciliary protein phosphorylation, we compared AC3 WT and KO datasets with Ciliome Database, a comprehensive ciliary proteome database maintained by the Leroux lab (http://www.sfu.ca/$\sim$leroux/ciliome_database.htm). Nine of the sixty five of proteins (14%) were listed in the Ciliome Database. Five of them (*Fam126a, Ggct, Pde1b, Ctnnd2*, and *Gapvd1)* were highly phosphorylated, whereas 4 (*Rabl6, Eif3b, Klc2*, and *Numbl*) exhibited lower phosphorylation in AC3 KOs. We did not find many hits (only 14%) that match with proteins that are listed in the Ciliome Database. We reasoned that our TiO_2_ enrichment method harvested phosphopeptides from whole tissue lysate of prefrontal cortex (mostly cytosol), and primary cilia or the basal body only contributed to a little portion of the peptide pool. Still, there are several interesting targets such as *Pde1b* (Li et al., 2004), *Rabl6* (Blacque et al., 2005)*, Klc2* (Li et al., 2004), and *Numbl* (Ramamurthy et al., 2014) that have been reported to be involved in ciliary biology.

To determine if some protein interaction network may be associated with the phosphoproteins differentially expressed in AC3 KOs and WTs, the 65 proteins were mapped onto the mouse Search Tool for the Retrieval of Interacting Genes/Proteins (STRING) database (string-db.org) of known protein interactions (Figure 5D). STRING is a Cytoscape-based protein-protein interaction network analysis software, which maps and predicts protein-protein interaction (Szklarczyk et al., 2015). AC3 (*Adcy3*) as well as PKA (*Prkaca*) were added into the STRING database manually to examine whether these 65 proteins also have direct or indirect interaction with each other. We found 34 of 65 proteins had direct or indirect interaction with each other (Figure 5D). Thirteen out of the thirty-four had decreased expression level in AC3 KOs, whereas 19 of them had increased expression in AC3 KOs. Two of them (*Map1a* and *Map2*) had both up-regulated and down-regulated phosphopeptides (Figure 5D). Notably, the protein interaction map centered around *Sptan1* (encoding α-II Spectrin). In the protein-protein interaction network, 45% of connectivity are established between *Sptan1* with other genes, removing *Sptan1* would dramatically change the network (Figure S4). *Sptan1* has a bi-phosphorylation site (S1186|T1188|S1190) detected in 4 AC3 KO samples, but in none of 8 WTs (see Table 2). *Sptan1* is a principal membrane skeleton component and provides a spectrin-actin cytoskeleton interface to integrate signals (Machnicka et al., 2012, 2014). *Sptan1* plays a critical role in neurodevelopment and mutations of *Sptan1* leads to encephalopathy, intellectual disability and ASD (Syrbe et al., 2017). Note that AC3 is associated with ASD (Skafidas et al., 2014; Yuen et al., 2017) and primary cilia regulate neurodevelopment (Valente et al., 2014).

### Differential Expression of Phosphopeptides in Male and Female Samples

Males and females differ greatly in cognition, behaviors, and disease susceptibility (Zagni et al., 2016). Remarkably, many psychiatric diseases, such as ADHD, MDD, and ASD, exhibit a sexual dimorphism (Zagni et al., 2016). But to date it is unclear what causes the sexual dimorphism of these disorders. We hypothesized that post-translational phosphorylation may correlate with the sexual dimorphism of ASD. To determine if there are any gender-biased phosphorylations in our samples, we compared female and male datasets. All phosphopeptides exclusively enriched (*p* < 0.05 by Two Populations Proportions comparison) in males or females are listed in Tables 3, 4. In total, 95 phosphorylated peptides (out of 90 proteins such as *Atp1a3, Srgap2*, and *Dlgap2*) were detected only in female samples, whereas 26 phosphopeptides (out of 26 proteins such as *Ctnnd1, Ctnnd2, Efnb3, and Caskin1*) were only found in male samples. Among these p-sites, one striking example is catenin δ-2 (p)Ser7, which was highly enriched in males. This site has been reported in male mice on UniPort database (Huttlin et al., 2010; UniProt Consortium, 2018). This p-site was detected in six out of eight male samples (3 KOs and 3 controls), but in none of the eight female samples, indicating that catenin δ-2 (p)Ser7 is a male-specific phosphorylate site. Notably, mutations in catenin δ-2 cause autism in female-enriched multiplex autism families (Turner et al., 2015)

**Table 3:** Phosphopeptides exclusively detected in female dataset.

**Gene**	**Protein name**	**Detection times (in females)**	**Peptide**	**References for ASD or related disorders**
Apc	Adenomatous polyposis coli protein	4	monophos-(1434)SKTPPPPPQTVQAK	Girirajan et al., 2013
Atp1a1	Sodium/potassium-transporting ATPase subunit alpha-1	6	monophos-(707)QGAIVAVTGDGVNDSPALKK	Schlingmann et al., 2018
Atp1a3	Sodium/potassium-transporting ATPase subunit alpha-3	6 6	monophos-(466)VAEIPFNSTNK monophos-(697)QGAIVAVTGDGVNDSPALKK	Myers et al., 2011 Mukaetova-Ladinska et al., 2004
Bin1	Myc box-dependent-interacting protein 1	3	monophos-(312)VNHEPEPASGASPGATIPK	Talkowski et al., 2012
Cnksr2	Connector enhancer of kinase suppressor of ras 2	3	monophos-(502)SNSPAHYSLLPSLQMDALR	Girirajan et al., 2013
Dlgap2	Disks large-associated protein 2	3	diphos-(1032)AASFRQNSATER	Myers et al., 2011
Jph3^*^	Junctophilin-3	5	monophos-(417)EFSPSFQHR	Mukaetova-Ladinska et al., 2004
Lrrc7	Leucine-rich repeat-containing protein 7	3	monophos-(1342)SREQQPYEGNINK	Talkowski et al., 2012
Magi2^*^	Membrane-associated guanylate kinase, WW and PDZ domain-containing protein 2	4	monophos-(1004)IIPQEELNSPTSAPSSEK	Girirajan et al., 2013
Map1b^*^	Microtubule-associated protein 1B	4	monophos-(1194)DYNASASTISPPSSMEEDKFSK	Myers et al., 2011
Map2	Microtubule-associated protein 2	3	monophos-(1004)ELITTKDTSPEK	Mukaetova-Ladinska et al., 2004
Map6^*^	Microtubule-associated protein 6	3	monophos-(293)SEGHEEKPLPPAQSQTQEGGPAAGK	Talkowski et al., 2012
Nav1^*^	Neuron navigator 1	3	monophos-(374)LELVESLDSDEVDLK	Girirajan et al., 2013
Neo1	Neogenin	3	monophos-(1200)LELKPIDKSPDPNPVMTDTPIPR	Myers et al., 2011
Plcd3^*^	1-phosphatidylinositol 4,5-bisphosphate phosphodiesterase delta-3	3	monophos-(489)ILSDREEEEEEEEEAEEALEAAEQR	Mukaetova-Ladinska et al., 2004
Prex1	Phosphatidylinositol 3,4,5-trisphosphate-dependent Rac exchanger 1 protein	3	monophos-(1178)SNSSYLGSDEMGSGDELPCDMR	Talkowski et al., 2012
Psmd4^*^	26S proteasome non-ATPase regulatory subunit 4	4	monophos-(237)AAAASAAEAGIATPGTEDSDDALLK	Girirajan et al., 2013
Ptpn1^*^	Tyrosine-protein phosphatase non-receptor type 1	4	monophos-(325)ELFSSHQWVSEETCGDEDSLAR	Myers et al., 2011
Slc12a6^*^	Solute carrier family 12 member 6	3	monophos-(21)IDDIPGLSDTSPDLSSR	Mukaetova-Ladinska et al., 2004
Smarcc1^*^	SWI/SNF complex subunit SMARCC1	3	diphos-(323)RKPSPSPPPPTATESR	Talkowski et al., 2012
Sorbs1^*^	Sorbin and SH3 domain-containing protein 1	6	monophos-(49)GTPSSSPVSPQESPKHESK	Girirajan et al., 2013
Spast	Spastin	3	diphos-(89)SSGTAPAPASPSPPEPGPGGEAESVR	Myers et al., 2011
Spry2^*^	Protein sprouty homolog 2	3	monophos-(108)SISTVSSGSR	Mukaetova-Ladinska et al., 2004
Srgap2^*^	SLIT-ROBO Rho GTPase-activating protein 2	5 3	monophos-(496)KQDSSQAIPLVVESCIR monophos-(692)GGSMEDYCDSTHGETTSAEDSTQDVTAEHHTSDDECEPIEAIAK	Talkowski et al., 2012 Girirajan et al., 2013
Srpk1^*^	SRSF protein kinase 1	44	monophos-(31)GSAPHSESDIPEQEEEILGSDDDEQEDPNDYCK monophos-(285)MQEIEEMEKESGPGQK	Myers et al., 2011 Mukaetova-Ladinska et al., 2004
Strip1^*^	Striatin-interacting protein 1	3	monophos-(56)KDSEGYSESPDLEFEYADTDK	Talkowski et al., 2012
Trio	Triple functional domain protein	3	monophos-(2274)NFLNALTSPIEYQR	Girirajan et al., 2013
Aagab	Alpha- and gamma-adaptin-binding protein p34	4	monophos-(196)VASAESCHSEQQEPSPTAER	#N/A
Aak1	AP2-associated protein kinase 1	4	monophos-(678)TSQQNVSNASEGSTWNPFDDDNFSK	#N/A
Acot11	Acyl-coenzyme A thioesterase 11	3	monophos-(24)SISHPESGDPPTMAEGEGYR	#N/A
Akap12	A-kinase anchor protein 12	4	diphos-(260)EKEPTKPLESPTSPVSNETTSSFK	#N/A
Amer2	APC membrane recruitment protein 2	4	monophos-(551)DSDSGDALCDLYVEPEASPATLPATEDPPCLSR	#N/A
Arfgef3	Brefeldin A-inhibited guanine nucleotide-exchange protein 3	3	monophos-(2050)GPDSPLLQRPQHLIDQGQMR	#N/A
Arhgef12	Rho guanine nucleotide exchange factor 12	3	monophos-(326)SEGVQDAEPQSLVGSPSTR	#N/A
Atg4c	Cysteine protease ATG4C	5	monophos-(424)DFDFTSTAASEEDLFSEDERK	#N/A
Atp1a2	Sodium/potassium-transporting ATPase subunit alpha-2	6	monophos-(474)VAEIPFNSTNK	#N/A
		6	monophos-(704)QGAIVAVTGDGVNDSPALKK	
Bsn	Protein bassoon	3	monophos-(1038)SHGPLLPTIEDSSEEEELREEEELLR	#N/A
Camkv	CaM kinase-like vesicle-associated protein	4	monophos-(395)SATPATDGSATPATDGSVTPATDGSITPATDGSVTPATDR	#N/A
Cir1	Corepressor interacting with RBPJ 1	3	monophos-(188)NLTANDPSQDYVASDCEEDPEVEFLK	#N/A
Cmtm4	CKLF-like MARVEL transmembrane domain-containing protein 4	4	monophos-(191)TESRDVDSRPEIQR	#N/A
Cpsf7	Cleavage and polyadenylation specificity factor subunit 7	3	monophos-(192)DSSDSADGRATPSENLVPSSAR	#N/A
Csnk1a1	Casein kinase I isoform alpha	3	monophos-(304)AAQQAASSSGQGQQAQTPTGK	#N/A
Dos	Protein Dos	3	monophos-(613)RGDSVDCPPEGR	#N/A
Epb41l1	Band 4.1-like protein 1	5	diphos-(465)SEAEEGEVRTPTK	#N/A
Epb41l3	Band 4.1-like protein 3	4	monophos-(48)QQPALEQFPEAAAHSTPVKR	#N/A
Evl	Ena/VASP-like protein	4	monophos-(232)VQRPEDASGGSSPSGTSK	#N/A
Farp1	FERM, RhoGEF and pleckstrin domain-containing protein 1	3	monophos-(387)QSPQSASLTFGEGTESPGGQSCQQAK	#N/A
Gpalpp1	GPALPP motifs-containing protein 1	3	monophos-(127)GREDPGQVSSFFNSEEAESGEDEDIVGPMPAK	#N/A
Hid1	Protein HID1	3	diphos-(583)TPEPLSRTGSQEGTSMEGSRPAAPAEPGTLK	#N/A
Hook3	Protein Hook homolog 3	4	monophos-(226)LNQSDSIEDPNSPAGR	#N/A
Hspa4	Heat shock 70 kDa protein 4	5	monophos-(521)MQVDQEEPHTEEQQQQPQTPAENKAESEEMETSQAGSK	#N/A
Ipo5	Importin-5	3	monophos-(814)RQDEDYDEQVEESLQDEDDNDVYILTK	#N/A
Kctd16	BTB/POZ domain-containing protein KCTD16	4	monophos-(282)WSSSHCDCCCK	#N/A
Lrrc47	Leucine-rich repeat-containing protein 47	3	monophos-(507)STSENKEEDMLSGTEADAGCGLSDPNLTLSSGK	#N/A
Lrsam1	E3 ubiquitin-protein ligase LRSAM1	6	monophos-(206)ESGLDYYPPSQYLLPVLEQDGAENTQDSPDGPASR	#N/A
Mvb12b	Multivesicular body subunit 12B	3	monophos-(194)NHDSSQPTTPSQSSASSTPAPNLPR	#N/A
Nckipsd	NCK-interacting protein with SH3 domain	5	monophos-(257)APSPEPPTEEVAAETNSTPDDLEAQDALSPETTEEK	#N/A
Ndel1	Nuclear distribution protein nudE-like 1	5	monophos-(223)GTENSFPSPK	#N/A
Nsf	Vesicle-fusing ATPase	3	monophos-(202)ENRQSIINPDWNFEK	#N/A
Nufip2	Nuclear fragile X mental retardation-interacting protein 2	4	monophos-(614)DYEIENQNPLASPTNTLLGSAK	#N/A
Ogfrl1	Opioid growth factor receptor-like protein 1	3	monophos-(372)EPGEEADKPSPEPGSGDPKPR	#N/A
Oxr1	Oxidation resistance protein 1	3	diphos-(358)QEKSSDASSESVQTVSQMEVQSLTATSEAANVPDR	#N/A
Pacsin1	Protein kinase C and casein kinase substrate in neurons protein 1	4	monophos-(388)ALYDYDGQEQDELSFK	#N/A
Pacsin3	Protein kinase C and casein kinase II substrate protein 3	6	monophos-(332)DGTAPPPQSPSSPGSGQDEDWSDEESPRK	#N/A
PAGR1	PAXIP1-associated glutamate-rich protein 1	3	monophos-(222)DLFSLDSEGPSPTSPPLR	#N/A
Pds5b	Sister chromatid cohesion protein PDS5 homolog B	3	monophos-(1353)AESPETSAVESTQSTPQK	#N/A
Pitpnc1	Cytoplasmic phosphatidylinositol transfer protein 1	3	diphos-(112)YEDNKGSNDSIFDSEAK	#N/A
Pnmal1	PNMA-like protein 1	3	monophos-(319)SALPAADSPGNLEDSDQDGGPENPAK	#N/A
Ppp1r7	Protein phosphatase 1 regulatory subunit 7	3	diphos-(20)RVESEESGDEEGK	#N/A
Prkce	Protein kinase C epsilon type	4	monophos-(343)SKSAPTSPCDQELK	#N/A
Psen1	Presenilin-1	3	monophos-(344)DSHLGPHRSTPESR	#N/A
Ptrf	Polymerase I and transcript release factor	4	diphos-(175)ESEALPEKEGDELGEGER PEDDTAAIELSSDEAVEVEEVIEESR	#N/A
Rad23a	UV excision repair protein RAD23 homolog A	4	diphos-(119)EDKSPSEESTTTTSPESISGSVPSSGSSGR	#N/A
Rap1gap2	Rap1 GTPase-activating protein 2	3	monophos-(8)KQELANSSDVTLPDRPLSPPLTAPPTMK	#N/A
Rasgrf2	Ras-specific guanine nucleotide-releasing factor 2	3	monophos-(722)KFSSPPPLAVSR	#N/A
Rps6kc1	Ribosomal protein S6 kinase delta-1	5	monophos-(646)ESEAQDSVSRGSDDSVPVISFK	#N/A
Rragc	Ras-related GTP-binding protein C	3	monophos-(83)MSPNETLFLESTNK	#N/A
Rtn1	Reticulon-1	5	diphos-(303)QDLCLKPSPDTVPTVTVSEPEDDSPGSVTPPSSG TEPSAAESQGK	#N/A
Serbp1	Plasminogen activator inhibitor 1 RNA-binding protein	3	monophos-(240)QISYNCSDLDQSNVTEETPEGEEHPVAD TENKENEVEEVK	#N/A
Snx16	Sorting nexin-16	3	monophos-(91)EAEEQHPEAVNWEDRPSTPTILGYEVMEER	#N/A
Ssbp3	Single-stranded DNA-binding protein 3	5	monophos-(345)NSPNNISGISNPPGTPR	#N/A
Stambpl1	AMSH-like protease	4	monophos-(232)SDGSNFANYSPPVNR	#N/A
Synpo	Synaptopodin	5	monophos-(760)VASLSPAR	#N/A
Tacc1	Transforming acidic coiled-coil-containing protein 1	3	monophos-(549)APVSVACGGESPLDGICLSEADK	#N/A
Tmf1	TATA element modulatory factor	4	monophos-(333)SVSEINSDDELPGK	#N/A
Tmpo	Lamina-associated polypeptide 2, isoforms beta/delta/epsilon/gamma	3	monophos-(60)GPPDFSSDEEREPTPVLGSGASVGR	#N/A
Trappc10	Trafficking protein particle complex subunit 10	4	monophos-(704)RQESGSSLEPPSGLALEDGAHVLR	#N/A
Trim28	Transcription intermediary factor 1-beta	3	monophos-(435)QGSGSSQPMEVQEGYGFGSDDPYSSAEPHVSGMK	#N/A
		4	diphos-(591)LASPSGSTSSGLEVVAPEVTSAPVSGPGILDDSATICR	
Tyro3	Tyrosine-protein kinase receptor TYRO3	3	monophos-(799)AEQPTESGSPEVHCGER	#N/A
Zfyve20	Rabenosyn-5	6	monophos-(206)DSLSTHTSPSQSPNSVHGSR	#N/A

Phosphopeptides listed in the table were detected in no <3 times in female dataset (n = 8), but never detected in any male dataset (n = 8). Proteins on top of the table were annotated in the AutDB (or per PubMed literature, labeled with

* *) to be associated with ASD or neurodevelopmental disorders. The number in brackets indicates the position of amino acid just before the peptide*.

**Table 4:** Phosphopeptides exclusively detected in male dataset.

**Gene**	**Protein name**	**Detection times (in Males)**	**Peptide**	**References for ASD or related disorders**
Abi1^*^	Abl interactor 1	3	monophos-(173)TNPPTQKPPSPPVSGR	Girirajan et al., 2013
Apc	Adenomatous polyposis coli protein	3	diphos-(1856)NDSLSSLDFDDDDVDLSR	Myers et al., 2011
Caskin1^*^	Caskin-1	3	monophos-(727)SQEYLLDEGMAPGTPPK	Mukaetova-Ladinska et al., 2004
Cspg5^*^	Chondroitin sulfate proteoglycan 5	3	monophos-(529)LKEEESFNIQNSMSPK	Talkowski et al., 2012
Ctnnd1^*^	Catenin delta-1	3	monophos-(344)GSLASLDSLRK	Girirajan et al., 2013
Ctnnd2	Catenin delta-2	6	monophos-(4)KQSGAAPFGAMPVPDQPPSASEK	Myers et al., 2011
Efnb3^*^	Ephrin-B3	3	monophos-(271)GGSLGLGGGGGMGPR	Mukaetova-Ladinska et al., 2004
Irf2bpl	Interferon regulatory factor 2-binding protein-like	3	diphos-(633)RNSSSPVSPASVPGQR	Talkowski et al., 2012
Map1b^*^	Microtubule-associated protein 1B	3	diphos-(1290)SVSPGVTQAVVEEHCASPEEK	Girirajan et al., 2013
Srgap2^*^	SLIT-ROBO Rho GTPase-activating protein 2	3	diphos-(981)TSPVVAPTSEPSSPLHTQLLKDPEPAFQR	Myers et al., 2011
Tbc1d5	TBC1 domain family member 5	3	monophos-(543)SESMPVQLNK	Mukaetova-Ladinska et al., 2004
Atat1	Alpha-tubulin N-acetyltransferase 1	3	diphos-(269)SSSLGNSPDRGPLRPFVPEQELLR	#N/A
Camsap1	Calmodulin-regulated spectrin-associated protein 1	4	diphos-(546)TDVSPPSPQMPR	#N/A
Cryab	Alpha-crystallin B chain	4	monophos-(56)APSWIDTGLSEMR	#N/A
Fam103a1	RNMT-activating mini protein	3	monophos-(31)RPPESPPIVEEWNSR	#N/A
Fam134a	Protein FAM134A	3	diphos-(293)TALALAITDSELSDEEASILESGGFSVSR	#N/A
Kbtbd11	Kelch repeat and BTB domain-containing protein 11	4	monophos-(60)ASAAEGSEASPPSLR	#N/A
Kiaa1467	Uncharacterized protein KIAA1467	6	monophos-(15)SPDLGEYDPLTQADSDESEDDLVLNLQQK	#N/A
Map4	Microtubule-associated protein 4	5	monophos-(514)DMSPSAETEAPLAK	#N/A
Mbp	Myelin basic protein	3	monophos-(145)YLATASTMDHAR	#N/A
Mrpl23	39S ribosomal protein L23, mitochondrial	5	monophos-(117)SPEPLEEELPQQR	#N/A
Phactr1	Phosphatase and actin regulator 1	4	diphos-(326)LESSEQRVPCSTSYHSSGLHSSDGITK	#N/A
Rbm5	RNA-binding protein 5	4	monophos-(614)GLVAAYSGDSDNEEELVER	#N/A
Sik3	Serine/threonine-protein kinase SIK3	3	monophos-(490)RASDGGANIQLHAQQLLK	#N/A
Slc6a17	Sodium-dependent neutral amino acid transporter SLC6A17	3	diphos-(679)VPSEAPSPMPTHR	#N/A
Sptbn1	Spectrin beta chain, non-erythrocytic 1	3	monophos-(2122)GDQVSQNGLPAEQGSPR	#N/A

Phosphopeptides listed in the table were detected in no <3 times in male dataset (n = 8), but never detected in any female dataset (n = 8). Proteins on top of the table were annotated in the AutDB (or per PubMed literature, labeled with

* *) to be associated with ASD or neurodevelopmental disorders. The number in brackets indicates the position of amino acid just before the peptide*.

**Figure 6:**
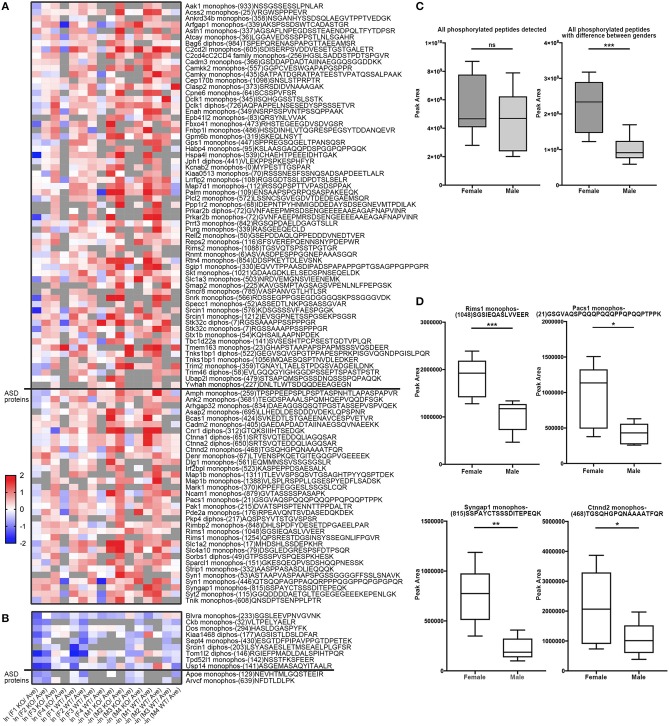
Comparison of phosphopeptide abundance in female and male datasets. **(A)** Heat map of 96 peptides (out of 88 proteins) having increased phosphorylation levels in females relative to males. Phosphopeptides with peak area of MS1 differed in males and females (*p* < 0.05, Student's *t*-test, unpaired) were included into the heatmap. 34 phosphopeptides from 31 Autism-related proteins were on the bottom whereas other 57 proteins were on the top. X-axis donates ln (peak area of individual peptide from female data/average peak area of all data) for female dataset, or –ln (peak area of individual peptide from male data/average peak area of all data) for male dataset. **(B)** Heat map of 11 peptides out of 11 proteins having increased phosphorylation levels in males relative to females. 2 phosphopeptides from 2 Autism-related proteins were on the bottom whereas other nine proteins were on the top. **(C)** The sum spectrum peak areas of all phosphopeptides have no difference in females (*n* = 8) and males (*n* = 8) (left). The sum spectrum peak areas of 107 phosphopeptides out of 99 proteins (as listed in **A,B**) were much higher in females than in males (*p* < 0.01, by unpaired Student's *t*-test). **(D)** Representative plots of four phosphopeptides (from *Rims1, Pacs1, Syngap1*, and *Ctnnd2)* of MS1-based quantification of phosphorylation levels in females compared to males.

Similarly, to compare phosphorylation level differences between females and males, Skyline-based label-free quantification was also performed on female and male datasets. We further found 96 phosphopeptides (out of 88 proteins) having increased phosphorylation levels in females relative to males, whereas only 11 peptides had significantly decreased phosphorylation levels in females (Figures 6A,B). Strikingly, 31 proteins (with 34 peptides) out of 88 (~35%) are associated with ASD, either annotated in the AutDB (Basu et al., 2009) or per PubMed reporting. Thirty-six of phosphopeptides out of the 33 proteins are presented in Figure 6B. Note that the overall phosphorylation levels had no differences between male and female samples, while the sum MS1 peak area of the 107 differentially expressed phosphopeptides were much higher in females than in males (Figure 6C). Phosphopeptide abundance of four representatives, *Rims1, Pacs1, Syngap1*, and *Ctnnd2* (both associated with ASD) are shown in Figure 6D. These data suggest that more proteins in females are highly phosphorylated in the frontal cortex than in males, with a high percentage of them being autism-associated proteins.

**Figure 7:**
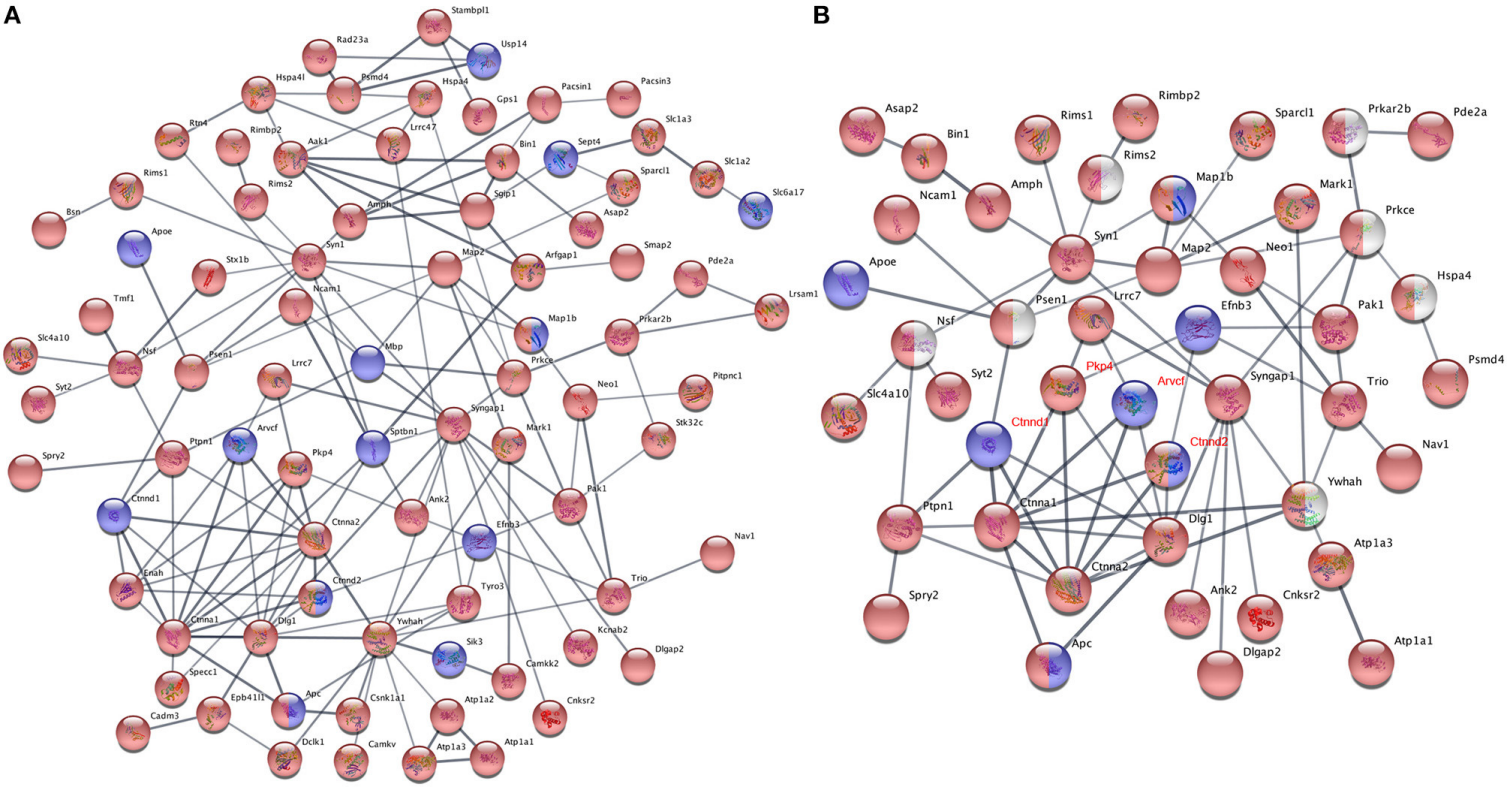
Protein-protein interaction analysis using phosphoproteins differentially expressed in females and males. **(A)** Proteins with phosphorylation differences between female and male dataset, which had presence-or-absence detection differences (listed in Tables 3, 4) or were identified in quantificational analysis (listed in Figures 6A,B), were mapped onto the mouse STRING database of protein-protein interactions. One hundred and ten of two hundred and four mapped proteins had interactions with each other, 82 of them were connected in a tight network, whereas other 28 proteins having interaction with a few proteins are not shown. The rest had no interaction with others. **(B)** STRING protein-protein interaction analysis of 63 ASD proteins with different phosphorylation levels in females and males. Thirty-eight of sixty-three proteins were connected in a tight interactions network with each other. Interaction score confidence = 0.400. For **(A,B)**, proteins with increased phosphorylation levels in females were highlighted in red and in males marked in blue. Proteins in red/blue had both increased and decreased p-sites on different positions in females and males. Proteins in white were manually added into the interaction map. The gene names of delta catenin family were written in red.

It is common that one protein activity can up- or down-regulate the activity of its interacting proteins in shared signal transduction pathways (von Mering et al., 2003). To assess if these ASD-related proteins interact with each other, 204 proteins with different phosphorylation levels in males or females (as listed Tables 3, 4 and Figures 6A,B) were mapped onto the mouse STRING database to examine protein-protein interaction. Figure 7A depicts that 82 out of 204 proteins were connected in a tight network, 69 proteins of the 82 had higher phosphorylation in females, three of them (e.g., *Ctnnd2*) had different phosphorylated peptides with increased phosphorylated levels in both females and males. Further, 63 proteins out of the 204 (~31%) with different phosphorylation levels in females or males were clearly associated with ASD. Thirty-two out of the sixty three of proteins were autism candidate entries in the AutDB, whereas the other 31 were reported in PubMed. It is noteworthy that all delta catenin proteins (*Ctnnd1, Ctnnd2, Pkp4*, and *Arvcf*, with names marked in red Figure 7B) exhibit gender-biased modifications. Furthermore, 50 out of the 63 ASD proteins had increased phosphorylation levels in female samples, whereas only eight proteins increased in male and five proteins had different phosphorylated peptides increased in both genders. STRING analysis further demonstrated that 38 of these 63 ASD proteins showed strong protein-protein interactions with each other (Figure 7B). These data demonstrate that 204 proteins had sex-biased phosphorylation and 31% of them were ASD-related genes which showed strong protein-protein interactions, suggesting that autism-related proteins may be highly regulated by post-translational phosphorylation in the female brain.

## Discussion

Malfunctions of primary cilia cause a broad spectrum of diseases in humans, particularly developmental disorders. AC3 is highly enriched in neuronal primary cilia, and not well-expressed in mature astrocyte cilia or microglia in the frontal cortex (Figure 1). AC3 is genetically associated with many human diseases including obesity (Nordman et al., 2008; Stergiakouli et al., 2014), ASD (Skafidas et al., 2014; Yuen et al., 2017), and MDD (Wray et al., 2012). It is unknown how AC3 regulates signaling network in central neurons. Besides activating the CNG channel in olfactory sensory neurons, AC3 (or cAMP) in olfactory cilia also sends signals to the cytosol of olfactory sensory neurons (DeMaria and Ngai, 2010) and regulates gene transcription (Serizawa et al., 2003). Given PKA is the major downstream protein of cAMP in most tissues, we postulated that identification of protein phosphorylation modulated by AC3 could help delineate AC3-regulated signaling network in central neurons.

We utilized a high-efficiency method to conduct comparative phosphoproteomics analyses combining TiO_2_ phosphopeptide enrichment with HPLC-MS/MS analysis. This approach allows for large-scale identification of phosphopeptides. In our assay, more than 1,500 phosphorylated peptides and 30,000 spectra were detected from each sample (Figure 3). We analyzed 16 samples and in total 4655 phosphopeptides were identified from 1756 proteins. We have manually verified all mass spectra presented in Figures 5–7 and Tables 1–4. This work provided a list of phosphoproteins that help elucidate the function of AC3 in the brain and unravel gender-biased protein phosphorylation. Intriguingly, we identified more gender-biased modifications than those of genotype-biased, suggesting that gender difference is much bigger than genotype differences in the frontal cortex in the phosphoproteomic assay.

To compare phosphorylation levels in proteins that are involved in cAMP signaling pathway, phosphopeptides identified from G-proteins, adenylyl cyclases, PKA, phosphodiesterases (PDE), phosphatases, as well as their regulating proteins were summarized in Table S4. It shows that phosphatases (such as *Ppp1r1b*) were highly phosphorylated, while G-protein α-subunits were not. cAMP-dependent protein kinases' catalytic subunits were less phosphorylated than their regulatory subunits (Table S4). Phosphorylation of two adenylyl cyclases (*Adcy5* and *Adcy9*) have been detected and apparently that *Adcy9* is highly phosphorylated in the frontal cortex. Among differentially expressed phosphopeptides between WTs and KOs, one interesting hit was *Pde1b* (p)Ser18. *Pde1b* is a calcium/calmodulin-dependent phosphodiesterase that breaks down both cAMP and cGMP (Sharma et al., 2006). *Pde1b* (p)Ser18 was detected in 4 of 8 AC3 KO mice (one female and three male animals), but in none of the control mice (Table 2), suggesting that *Pde1b* may function downstream of AC3. Additionally, *Ppp1r14A* (p)Ser19 (protein phosphatase 1 regulatory subunit 14A), which was detected in 4 of 8 WT mice (2 female and 2 male animals), but in none of 8 KO mice (Table 1). *Ppp1r14A* is a C-kinase phosphorylation-dependent inhibitor protein of phosphatase, implicated in cerebellar long-term synaptic plasticity (Eto et al., 2002). In contrast, *Ppp1r1b*, which is a cAMP/PKA-dependent phosphoprotein and regulates the activity of phosphatase-1, had at least four sites identified (Ser42|45|46, Ser97, Ser130, Thr182|192) in the dataset. However, we did not detect significant differences in phosphorylation levels in these four sites between WT and KO datasets (Table S4). Additionally, both Rho GTPase-activating protein 20 (*Arhgap20*) and Rho GTPase-activating protein 44 (*Arhgap44*) have p-sites that were exclusively detected in KO dataset (Table 2), suggesting these RhoGaps may be regulated by AC3 in the frontal cortex.

Male and female brains differ in many aspects including connectivity (Ingalhalikar et al., 2014), disease susceptibility (Zagni et al., 2016), and gene expression (Trabzuni et al., 2013). Numerous studies have attempted to use different approaches including next-generation sequencing technology to decipher the differences between male and female brains (Trabzuni et al., 2013; Werling et al., 2016; Gershoni and Pietrokovski, 2017). However, current research apparently has overlooked posttranslational modification differences in male and female brains, and phosphoproteomics databanks (UnitProt and Phosphosites) thus far have not collected any data specifically from female brain samples. This study filled this gap and we have conducted systematic phosphoproteomic profiling using prefrontal cortical samples of both genders. Consequently, we have identified 95 phosphopeptides only present in female samples, and 26 phosphopeptides restricted to male samples (Tables 3, 4). Label-free mass spectrometric quantification further revealed that 96 phosphopeptides have higher phosphorylation levels in females, while 11 phosphopeptides are more abundantly expressed in males. We found that phosphorylation of many autism-associated proteins, including but not limited to *Dlg1, Dlgap2, Syn1, SynGap1*, and *Srgap2* (Figures 6, 7 and Tables 3, 4), are gender-biased, occurring more in females than in males. As shown in Figure 6B and Tables 3, 4, 63 out of 204 phosphopeptides (~31%) are from autism-associated proteins. Some proteins/genes are not listed in the AutDB, but they directly interact with autism proteins or were found to be associated with autism per literature in PubMed. For example, *Caskin1* Ser 728 was only identified in males (Table 4). Caskin-1 interacts with neurexins, which bind to neuroligins in the synapses. Both neurexins and neuroligins are strongly associated with autisms (Südhof, 2017). *Caskin1* itself is implicated in autism (Daimon et al., 2015).

Among all differentially modified proteins in genders, one protein family is of particular interest. That is the delta catenin family, which contains ARVCF (encoded by *Arvcf*), catenin δ-1 (*Ctnnd1*), catenin δ-2 (*Ctnnd2*), and plakophilin-4 (*Pkp4*) (Yuan and Arikkath, 2017). All the delta catenin proteins are expressed in the central nervous system and regulate neural development, and they are strongly implicated in neurodevelopmental disorders (Turner et al., 2015; Yuan and Arikkath, 2017). Remarkably, the activity of delta catenin family is highly regulated by post-translational modifications such as ubiquitination and phosphorylation to modulate protein-protein interaction with cadherin, membrane localization, and protein stability (Yuan and Arikkath, 2017). Of note, this family has two regions flanking the ARM domain that are highly enriched with phosphorylation sites (Yuan and Arikkath, 2017). Interestingly, all four delta catenin proteins: ARVCF (Suzuki et al., 2009), catenin δ-1 (Hussman et al., 2011), catenin δ-2 (Turner et al., 2015), and plakophilin-4 (Hussman et al., 2011) are implicated in ASD or neurodevelopmental disorders. Relevant to the sexual dimorphism of autism, mutations of catenin δ-2 has been found to cause severe autism in female-enriched multiplex autism families (Turner et al., 2015). Loss-of-function of catenin δ-2 causes severe developmental phenotypes in animal model. Our MS analysis revealed that catenin δ-2 Ser 7 was restricted to males. Moreover, catenin δ-2 Thr 469 (another p-site) have increased phosphorylation in females relative to males. We have also detected p-sites of the delta catenin family which are also differentially modified in males or females: catenin δ-1 Ser 346 was only detected in males (Table 4); *ARVCF* Thr 643 has decreased phosphorylation in females; *PKP4* Ser 220 had higher expression in females (Figure 6). Together, these data suggest that the delta catenin family may participate in the regulation of gender-biased posttranslational phosphorylations, consequently affecting neuronal development in the prefrontal cortex. This interpretation is further supported by STRING analysis, showing that these four proteins are connected in a String interaction map with gender-biased phosphorylation (Figure 7B).

In summary, this comparative phosphoproteomic profiling has generated several interesting findings: (1) AC3 ablation leads to decreased activity of proline-directed kinases in the frontal cortex; (2) There is a gender-biased phosphorylation in 204 proteins, 31% of which are associated with ASD; (3) Four delta catenin family members, all associated with autism, contain gender-biased phosphorylation sites. Hence, although future work is warranted, this study provides useful phosphoproteomic clues to elucidate the function of AC3 in the CNS. It also presents the first proteomic evidence suggesting that sex-biased post-translational phosphorylation is implicated in the sexual dimorphism of autism.

## Data Deposition

Proteome raw and complete datasets (4 groups: AC3 controls, KOs, males and females, 16 mice) have been submitted to ProteomeXchange via the PRIDE-EBI database (PXD012259).

## Ethics Statement

All animal-related procedures were approved and conducted in accordance with the guidelines of the Institutional Animal Care and Use Committee of the University of New Hampshire.

## Author Contributions

YZ and XC designed experiments. YZ conducted phosphoproteomic assays, immunostaining, and data analysis. LQ maintained mouse colony and provided support for phosphoproteomic assay. AS contributed to immunostaining. FC provided expertise in phosphoproteomic experiments and data analysis. HW provided expertise in statistical methods. YZ and XC interpreted data and wrote the paper.

### Conflict of Interest Statement

The authors declare that the research was conducted in the absence of any commercial or financial relationships that could be construed as a potential conflict of interest.
