# Supplementary material for: Comparative Phosphoproteomic Profiling of Type III Adenylyl Cyclase Knockout and Control, Male, and Female Mice
Source: Front Cell Neurosci. 2019 Feb 13;13:34. doi: 10.3389/fncel.2019.00034 (PMC6381875; doi:10.3389/fncel.2019.00034)
Supplement: Supplementary file 5 [file Data_Sheet_1.pdf]

Supplemental Information (SI)

Supplemental Figure Legends

Fig. S1. Western blot validation. **(A)** Representative Western blot images (n=2 biological replicas plus 2 technical replicas). **(B-E)** The empty bars showed the relative quantitative densitometry of phosphorylation level, which was normalized to the loading control GADPH. The phosphorylation levels of Syn1 p-Ser 605, CaMK2a p-Thr286, Erk2 pT203/205 and Erk1 pT183/185 didn't have significant differences between AC3 WT and KO mice, or between female and male mice. Grey bars were constructed by quantification data of MS1 spectra peak area of Syn1 p-Ser 605, CaMK2a p-Thr286, Erk2 pT203/205 and Erk1 pT183/185 phosphopeptides. There was no differences between KOs and WTs, or between females and males (n=16, 8 KOs, 8 WTs), which was consistent with Western blot results.

Fig. S2. Gene ontology enrichment analysis. Gene Ontology (GO) of biological process, cellular component, molecular function, Panther pathways, and protein class that were enriched in our dataset. Figures were constructed by proteins which were enriched in the dataset compared to GO annotation mouse genome database.

Fig. S3. Workflow for Motif-X analysis. Motif-X (i.e. Motif Extractor) is a bioinformatic tool designed to extract overrepresented motif patterns from a dataset. After a dataset, all the phosphopeptides detected in one of 16 samples, is uploaded to the Motif-X website (http://motif-x.med.harvard.edu/), the software first finds the 'central residue', (p)Ser, and elongates 6 residues on both sides (upstream and downstream). If less than 6 residues are present on one side of the peptide, it then searches the database to find a sequence that matches this phosphopeptide's sequence to 6 residues on both sides. It repeats the searching steps for all phosphopeptides in the dataset. Consequently, some common motifs will be matched by numerous peptides and are then identified to be enriched in this dataset. One kinase will recognize a specific motif pattern in order to fulfill phosphorylation, therefore the motif enrichment levels in a dataset can reflect the kinase activity in the sample. Similar analysis repeated to other 15 samples. This software generated "the fold increase", which means the enrichment level of the extracted motifs. The fold increase of specific motifs generated basing on 16 samples were used to do further statistical analysis.

Fig. S4. Protein-protein interaction analysis using phosphoproteins differentially expressed in AC3 KOs and WTs, without *Sptan1*. AC3 and PKA were manually included whereas Sptan1 was manually removed. Phosphoproteins with increased phosphorylated levels in KOs were highlighted in red, or in WTs highlighted in blue. Proteins marked in half red/blue had both increased and decreased p-sites on different positions in both WTs and KOs. Interaction score confidence=0.400. The STRING analysis including *Sptan1* using proteins having phosphorylation differences between AC3 KO and WT generated a network with 36 nodes and 49 edges, whereas STRING analysis without Sptan1 generated a network with 30 nodes and 27 edges, 45% of protein-protein interactions lost because of *Sptan1* ablation. None of other genes have a similar number of interactions.

Supplemental Materials and Methods

Mice

All animal-related procedures were approved and conducted in accordance with the guidelines of the Institutional Animal Care and Use Committee of the University of New Hampshire. The AC3 floxed mouse strain was generated as previously reported (Chen et al., 2016), and cross-bred with UBC-Cre/ERT2. The mouse genetic background was C57Bl/6j. DNA primers used to genotype AC3 floxed mice were forward ACCCTTTGAGGCCAGGGGCAA, reverse CTGCGGTGAGAGCCTGGCACA (Chen et al., 2016). Mice were maintained on a 12-h light/dark cycle at 22°C and had access to food and water *ad libitum*. AC3 flox/flox:Ubc-cre /ERT2 mice (mixed genders, female: male=1:1) were given an oral gavage of tamoxifen for AC3 KO mice (0.2mg/g body weight, 7 days) or vehicle (corn oil) for WT control mice at the age of 8-10 weeks to induce Cre recombinase expression to ablate AC3 ubiquitously. To verify that AC3 was efficiently deleted in mice, we quantified the efficiency of inducible AC3 ablation with immunostaining. Without Cre-expression induction (controls), 49.3± 5.0% cells (n = 3 animals) in the cortex possessed AC3-positive signal in primary cilia (note: astrocytes and microglia generally do not express AC3), while after tamoxifen injection, only 1.5± 1.2 % cells (n = 2 animals) had AC3-positive signal, indicating that AC3 in more than 95% neurons was successfully delated in KOs.

Immunofluorescence Staining and Confocal Imaging

AC3 KO and control mice were euthanized and perfused with PBS followed by 4% fresh paraformaldehyde (PFA). Isolated brain tissue was post-fixed in 4% fresh PFA in 4ºC overnight and dehydrated in 30% sucrose for 48 hours or until precipitation. Fixed brain tissue was flash-frozen in dry ice and embedded with tissue-tek® optimal cutting compound (O.C.T.). Tissues were sliced into 30-40 μm slices in -17ºC with a Shandon Cryostat. Tissue was then permeabilized for 10 minutes on an orbital shaker in PBST (PBS with 0.5% Triton). Blocking occurred in room temperature for 1-2 hours with 2 mg/ml BSA (Sigma, A9647), 0.1 M glycine, and 10% of donkey serum (Sigma, D9663) in PBS. Brain slices were stained with anti-ACIII antibody (EnCor Biotechnology, AB2572219, 1:10,000 dilution), anti-GFAP (Agilent, Z0334, 1:500 dilution), IBA1 (Abcam, ab5076, 1:1,000 dilution), and NeuN (Millipore, MAB377, 1:1,000 dilution) overnight at 4°C. Following primary antibody incubation, sections were washed 3 times for 10 minutes with PBS with 0.2% Triton X-100, and then incubated for 2 hours at room temperature with secondary antibodies from Thermo Fisher Scientific, including Alexa Fluors: 546, 647, and 488 at 1:500 dilution each. Nuclei were stained with Hoechst 33342 (0.1 μg/ml) or DAPI (1 μg/ml) for 5 min in room temperature. Sections were mounted with Southern Biotech Fluoromount-G and sealed with clear nail polish. Fluorescence images were acquired by Nikon A1R HD confocal Ti2 inverted microscope.

Western Blot

Cortex tissue extractions were resolved in 9% homemade SDS-PAGE. Proteins were wet-transferred onto PVDF membrane (0.2 μm, Cat. # 162-0177, Bio-Rad, Hercules, CA) and stained with rabbit anti-CaMKII pT286 (1:2000, Cell Signaling, 12716), anti-Syn1 pS605 (1:2000, Cell Signaling, 88246), anti-ERK2 pT203/Y205, pERK1 pT183/Y185 (1:1000, Cell Signaling, 4370). Anti-GAPDH (1:10000, Cell Signaling, 5174) was used as an internal control for normalizing sample loading. Primary antibodies were diluted in 5% BSA/TBS and incubated overnight a 4ºC.

Anti-rabbit IgG, HRP-linked Antibody (1:3000, Cell Signaling, 7074) was used as a secondary antibody for enhanced chemiluminescence.

Statistics to determine "more female-biased phosphorylation than male-biased".
(1) "Two Population Proportions" comparison. Using the 3 out of the n=8 sample cut-off method and label-free spectrum quantification method, we have identified 191 and 37 phosphopeptides that were enriched in the female and the male group, respectively. We identified a total of 2427 and 2158 phosphopeptides present in at least 3 biological replicas of the female group and the male group, respectively. The proportion of female biased phosphorylation in the female pool was 191/2427 =7.9%, while the male-biased phosphorylation proportion was 37/2158=1.7%. Using this comparison method, we calculated a z-score=9.87, p-value <0.0001, suggesting that these two proportions were significantly different.

(2) "Student t-test", We first counted how many sex-biased phosphopeptides present in each sample and calculated their ratio relative to the number of phosphopeptides (which were present in at least 3 replicas) in one sample, repeated this calculation for n=8 samples for males and females, respectively. For example, ($\frac{148\ of\ 228\ sex{-}biased\ petides\ deteted\ in\ F\ KO1}{1903\ of\ 2748\ peptides\ detected\ in\ 3\ or\ more\ samples\ in\ at\ least\ 1\ group\ deteted\ in\ F\ KO1} = 7.78\%$). The ratios were (5.72%, 5.73%, 4.59%, 4.77%, 5.28%, 6.11%, 5.05%, 4.87%) and (7.78%, 7.64%, 7.57%, 4.11%, 8.57%, 8.40%, 6.98%, 4.27%) in male and female groups, respectively. Conducting an unpaired Student t-test yielded a p-value=0.023<0.05. Together, both statistical analyses supported the conclusion that there are more female-biased phosphorylations than those of male-biased.

Statistics to determine "a high percentage of the sex-biased phosphoproteins are from ASD-associated genes"
There is no specific information regarding how many genes are ASD genes or what is the percentage of ASD genes in human genome. However, AutDB (Autism Gene Database, a useful and comprehensive resource for ASD research) collects a total of 1053 ASD gene entries (as of September 2018). The human genome is estimated to have 20,000 genes, thus all AutDB ASD genes are estimated to represent 5.2% (1053/20000 =0.052) of all human genes in the human genome. Regardless of additional autism reports in the PubMed, among the 204 differentially expressed peptides in male and female datasets (sex-biased modifications), we have found 32 proteins that are also listed in the AutDB as ASD genes. The ASD gene percentage is 32/204=15.6%, which was much higher than 5.2%. Statistically, we also used "Two Population Proportions" method to compare two groups (proportion of autism genes in human genome Vs. proportion of autism genes in sex-biased phosphoproteins). This produced a Z-score = 6.57 and p-value <0.0001, suggesting that two percentages (5% Vs 15.5%) were significantly different. Please note that the sample size would affect z-score or p-value of "Two Population Proportions" comparison drastically. Both AutDB has collected a great number of ASD genes and we also identified many sex-biased phosphoproteins and ASD genes, which allowed us to statistically make this comparison and support our conclusion with confidence. Although it was not a perfect statistical comparison, this statistical calculation supports the conclusion that a high percentage of the sex-biased phosphoproteins are from ASD-associated genes.

Statistics of Cross Comparison of 4 groups

The cross comparison of 4 groups requires the comparison of four proportions, so we used Pearson's Chi-square test to test the null hypothesis of $p_1 = p_2 = p_3 = p_4$ against the alternative that they were not all equal. Take the case of (4,0,0,0) as an example. Denoted $O_1 = 4,\ O_2 = O_3 = O_4 = 0$, and calculated $E_1 = E_2 = E_3 = E_4 = \frac{4+0+0+0}{4} = 1$. The Chi-square test statistics was

$$\chi^2 = \sum_{i=1}^{4} \frac{(O_i - E_i)^2}{E_i} = \frac{(4-1)^2}{1} + \frac{(0-1)^2}{1} + \frac{(0-1)^2}{1} + \frac{(0-1)^2}{1} = 12$$

The p-value was calculated from a Chi-square distribution with degrees of freedom 3, $\chi^2(3)$, p-value= $P(\chi^2(3)\ > \chi^2) = 0.00738 < 0.05$. Other p-values in the table were calculated in the same way.

(x_1, x_2, x_3, x_4)	p-value
(4 ,0 ,0 ,0)	0.00738
(4, 1, 0, 0)	0.035
(4, 2, 0, 0)	0.062
(3, 0, 0, 0)	0.029
(3, 1, 0, 0)	0.1
(4, 4, 0, 0)	0.046

According to this table, the cut-off is changed depending on whether a phosphorylation site was detected in 3 times in 4 samples in one group and wasn't detected in any sample from the other 3 groups, or it was detected in all 4 samples in one group, 1 or 4 times in another group and wasn't detected in any sample from other 2 groups. If p-value < 0.05, this phosphorylation site can be identified as a site with difference among the 4 groups. We have conducted a cross comparison of 4 groups (Female KOs, Females WTs, Male KOs, Male WTs) using Pearson's Chi-square test. Results are shown in the Supplemental Table below. We did not find many hits or clues from the Cross Comparison of 4 groups, which has persuaded us from further increasing sample size.

Supplemental Table: Results of Cross comparison of 4 groups (WT males; WT females; KO males; KO females).

Gene	Protein name	Peptide	Detection times in			
			F KO	F WT	M KO	M WT
Nav1	Neuron navigator 1	monophos-(374)LELVESLDSDEVDLK	3	0	0	0
Serbp1	Plasminogen activator inhibitor 1 RNA-binding protein	monophos-(240)QISYNCSDLDQSNVTEE TPEGEEHPVADTENK	3	0	0	0
Tyro3	Tyrosine-protein kinase receptor TYRO3	monophos-(799)AEQPTESGSPEVHCGER	3	0	0	0
Map2	Microtubule-associated protein 2	monophos-(1004)ELITTKDTSPEK	0	3	0	0
Spast	Spastin	diphos-(89)SSGTAPAPASPSPPEPGPGGE AESVR	0	3	0	0

Synpo	Synaptopodin	monophos-(760)VASLSPAR	4	1	0	0
Hepacam	Hepatocyte cell adhesion molecule	monophos-(316)DKDSSEPDENPATEPR	4	0	1	0
Mrpl23	39S ribosomal protein L23, mitochondrial	monophos-(117)SPEPLEEELPQQR	0	0	1	4

Choosing appropriate statistical methods for data comparison:

In this manuscript, we mostly used Two Population Proportions comparison (3 out of n=8 cut off) as well as Student's t-test for data comparison. An alternative comparison method was to separately compare two groups (i.e. Female KOs Vs. Female WTs, Male KOs Vs. Male WTs, and so on) for four times. However, this would dramatically decrease the sample size of each group from 8 to 4. Small sample size would reduce statistical power. Additionally, the statistical cut-off would be changed to the "3 out of n=4" p-value=0.014, not the "2 out of n =4" p-value=0.079 (according to Two Population Proportions comparison). The high cut-off would lead to loss of positive information. Moreover, if a phosphorylation site was detected 4 times in 4 F WTs and 0 time in 4 M WT, but also detected once in both 4 F KOs and 4 M KOs (4, 0, 1, 1), we would be unable to determine whether this site was enriched in female or it showed no difference between genders. Thus we did not use this method.

The target gene *Adcy3* is localized on euchromosome (Human 2p23.3, Mouse Chr12A-B), not sex chromosome. We have studied AC3 for eight years and thus far we have not observed gender differences of AC3 expression in olfactory cilia and neuronal primary cilia. AC3 is expressed in both males and females. Both conventional and conditional AC3 knockout mice lead to similar effects in males and females (such as loss of smell, obesity, depression-like phenotypes, as well as other phenotypes). Statistically, gender can be considered a random variable when comparing genotypes. Similarly, the genotype can be considered a random variable when comparing genders. Together, we chose to compare our results by "WTs Vs. KOs (n=8 pairs)" and "Females Vs. Males (n=8 pairs)" using Two Population Proportions comparison as well as Student's t-test.

Additional References cited in the Tables:

Barber, J. C., Ellis, K. H., Bowles, L. V., Delhanty, J. D., Ede, R. F., Male, B. M., & Eccles, D. M. (1994). Adenomatous polyposis coli and a cytogenetic deletion of chromosome 5 resulting from a maternal intrachromosomal insertion. *J Med Genet, 31*(4), 312-316.

Campbell, I. M., Yatsenko, S. A., Hixson, P., Reimschisel, T., Thomas, M., Wilson, W., . . . Scaglia, F. (2012). Novel 9q34.11 gene deletions encompassing combinations of four Mendelian disease genes: STXBP1, SPTAN1, ENG, and TOR1A. *Genet Med, 14*(10), 868-876. doi:10.1038/gim.2012.65

Carlisle, H. J., Luong, T. N., Medina-Marino, A., Schenker, L., Khorosheva, E., Indersmitten, T., . . . Kennedy, M. B. (2011). Deletion of densin-180 results in abnormal behaviors associated with mental illness and reduces mGluR5 and DISC1 in the postsynaptic density fraction. *J Neurosci, 31*(45), 16194-16207. doi:10.1523/JNEUROSCI.5877-10.2011

Connolly, J. J., Glessner, J. T., & Hakonarson, H. (2013). A genome-wide association study of autism incorporating autism diagnostic interview-revised, autism diagnostic observation schedule, and social responsiveness scale. *Child Dev, 84*(1), 17-33. doi:10.1111/j.1467-8624.2012.01838.x

Correia, C., Oliveira, G., & Vicente, A. M. (2014). Protein interaction networks reveal novel autism risk genes within GWAS statistical noise. *PLoS One, 9*(11), e112399. doi:10.1371/journal.pone.0112399

Daimon, C. M., Jasien, J. M., Wood, W. H., 3rd, Zhang, Y., Becker, K. G., Silverman, J. L., . . . Maudsley, S. (2015). Hippocampal Transcriptomic and Proteomic Alterations in the BTBR Mouse Model of Autism Spectrum Disorder. *Front Physiol, 6*, 324. doi:10.3389/fphys.2015.00324

De Rubeis, S., He, X., Goldberg, A. P., Poultney, C. S., Samocha, K., Cicek, A. E., . . . Buxbaum, J. D. (2014). Synaptic, transcriptional and chromatin genes disrupted in autism. *Nature, 515*(7526), 209-215. doi:10.1038/nature13772

Deciphering Developmental Disorders, S. (2017). Prevalence and architecture of de novo mutations in developmental disorders. *Nature, 542*(7642), 433-438. doi:10.1038/nature21062

Demos, M. K., van Karnebeek, C. D., Ross, C. J., Adam, S., Shen, Y., Zhan, S. H., . . . Consortium, F. C. (2014). A novel recurrent mutation in ATP1A3 causes CAPOS syndrome. *Orphanet J Rare Dis, 9*, 15. doi:10.1186/1750-1172-9-15

Dennis, M. Y., Nuttle, X., Sudmant, P. H., Antonacci, F., Graves, T. A., Nefedov, M., . . . Eichler, E. E. (2012). Evolution of human-specific neural SRGAP2 genes by incomplete segmental duplication. *Cell, 149*(4), 912-922. doi:10.1016/j.cell.2012.03.033

Girirajan, S., Dennis, M. Y., Baker, C., Malig, M., Coe, B. P., Campbell, C. D., . . . Eichler, E. E. (2013). Refinement and discovery of new hotspots of copy-number variation associated with autism spectrum disorder. *Am J Hum Genet, 92*(2), 221-237. doi:10.1016/j.ajhg.2012.12.016

Hussman, J. P., Chung, R. H., Griswold, A. J., Jaworski, J. M., Salyakina, D., Ma, D., . . . Pericak-Vance, M. A. (2011). A noise-reduction GWAS analysis implicates altered regulation of neurite outgrowth and guidance in autism. *Mol Autism, 2*(1), 1. doi:10.1186/2040-2392-2-1

Hwang, J., & Pallas, D. C. (2014). STRIPAK complexes: structure, biological function, and involvement in human diseases. *Int J Biochem Cell Biol, 47*, 118-148. doi:10.1016/j.biocel.2013.11.021

Kanduri, C., Kantojarvi, K., Salo, P. M., Vanhala, R., Buck, G., Blancher, C., . . . Jarvela, I. (2016). The landscape of copy number variations in Finnish families with autism spectrum disorders. *Autism Res, 9*(1), 9-16. doi:10.1002/aur.1502

Kuhnle, S., Martinez-Noel, G., Leclere, F., Hayes, S. D., Harper, J. W., & Howley, P. M. (2018). Angelman syndrome-associated point mutations in the Zn-binding N-terminal (AZUL) domain of UBE3A ubiquitin ligase inhibit binding to the proteasome. *J Biol Chem*. doi:10.1074/jbc.RA118.004653

Li, J., Chai, A., Wang, L., Ma, Y., Wu, Z., Yu, H., . . . Zhang, D. (2015). Synaptic P-Rex1 signaling regulates hippocampal long-term depression and autism-like social behavior. *Proc Natl Acad Sci U S A, 112*(50), E6964-6972. doi:10.1073/pnas.1512913112

Liu, X., Shimada, T., Otowa, T., Wu, Y. Y., Kawamura, Y., Tochigi, M., . . . Gau, S. S. (2016). Genome-wide Association Study of Autism Spectrum Disorder in the East Asian Populations. *Autism Res, 9*(3), 340-349. doi:10.1002/aur.1536

Liu, Y. F., Sowell, S. M., Luo, Y., Chaubey, A., Cameron, R. S., Kim, H. G., & Srivastava, A. K. (2015). Autism and Intellectual Disability-Associated KIRREL3 Interacts with Neuronal Proteins MAP1B and MYO16 with Potential Roles in Neurodevelopment. *PLoS One, 10*(4), e0123106. doi:10.1371/journal.pone.0123106

Lopez-Hernandez, T., Ridder, M. C., Montolio, M., Capdevila-Nortes, X., Polder, E., Sirisi, S., . . . van der Knaap, M. S. (2011). Mutant GlialCAM causes megalencephalic leukoencephalopathy with subcortical cysts, benign familial macrocephaly, and macrocephaly with retardation and autism. *Am J Hum Genet, 88*(4), 422-432. doi:10.1016/j.ajhg.2011.02.009

Lovrecic, L., Bertok, S., & Zerjav Tansek, M. (2016). A New Case of an Extremely Rare 3p21.31 Interstitial Deletion. *Mol Syndromol, 7*(2), 93-98. doi:10.1159/000445227

Marshall, C. R., Noor, A., Vincent, J. B., Lionel, A. C., Feuk, L., Skaug, J., . . . Scherer, S. W. (2008). Structural variation of chromosomes in autism spectrum disorder. *Am J Hum Genet, 82*(2), 477-488. doi:10.1016/j.ajhg.2007.12.009

Moser, D., Ekawardhani, S., Kumsta, R., Palmason, H., Bock, C., Athanassiadou, Z., . . . Meyer, J. (2009). Functional analysis of a potassium-chloride co-transporter 3 (SLC12A6) promoter polymorphism leading to an additional DNA methylation site. *Neuropsychopharmacology, 34*(2), 458-467. doi:10.1038/npp.2008.77

Mukaetova-Ladinska, E. B., Arnold, H., Jaros, E., Perry, R., & Perry, E. (2004). Depletion of MAP2 expression and laminar cytoarchitectonic changes in dorsolateral prefrontal cortex in adult autistic individuals. *Neuropathol Appl Neurobiol, 30*(6), 615-623. doi:10.1111/j.1365-2990.2004.00574.x

Myers, R. A., Casals, F., Gauthier, J., Hamdan, F. F., Keebler, J., Boyko, A. R., . . . Awadalla, P. (2011). A population genetic approach to mapping neurological disorder genes using deep resequencing. *PLoS Genet, 7*(2), e1001318. doi:10.1371/journal.pgen.1001318

Ogiwara, I., Miyamoto, H., Tatsukawa, T., Yamagata, T., Nakayama, T., Atapour, N., . . . Yamakawa, K. (2018). Nav1.2 haplodeficiency in excitatory neurons causes absence-like seizures in mice. *Commun Biol, 1*. doi:10.1038/s42003-018-0099-2

Pettersson, M., Viljakainen, H., Loid, P., Mustila, T., Pekkinen, M., Armenio, M., . . . Lindstrand, A. (2017). Copy Number Variants Are Enriched in Individuals With Early-

Onset Obesity and Highlight Novel Pathogenic Pathways. *J Clin Endocrinol Metab, 102*(8), 3029-3039. doi:10.1210/jc.2017-00565
Pinto, D., Pagnamenta, A. T., Klei, L., Anney, R., Merico, D., Regan, R., . . . Betancur, C. (2010). Functional impact of global rare copy number variation in autism spectrum disorders. *Nature, 466*(7304), 368-372. doi:10.1038/nature09146
Sanders, S. J., He, X., Willsey, A. J., Ercan-Sencicek, A. G., Samocha, K. E., Cicek, A. E., . . . State, M. W. (2015). Insights into Autism Spectrum Disorder Genomic Architecture and Biology from 71 Risk Loci. *Neuron, 87*(6), 1215-1233. doi:10.1016/j.neuron.2015.09.016
Schlingmann, K.P., Bandulik, S., Mammen, C., Tarailo-Graovac, M., Holm, R., Baumann, M., et al. (2018). Germline De Novo Mutations in ATP1A1 Cause Renal Hypomagnesemia, Refractory Seizures, and Intellectual Disability. *Am J Hum Genet* 103(5)**,** 808-816. doi: 10.1016/j.ajhg.2018.10.004.
Siu, W. K., Lam, C. W., Gao, W. W., Vincent Tang, H. M., Jin, D. Y., & Mak, C. M. (2016). Unmasking a novel disease gene NEO1 associated with autism spectrum disorders by a hemizygous deletion on chromosome 15 and a functional polymorphism. *Behav Brain Res, 300*, 135-142. doi:10.1016/j.bbr.2015.10.041
Stamova, B. S., Tian, Y., Nordahl, C. W., Shen, M. D., Rogers, S., Amaral, D. G., & Sharp, F. R. (2013). Evidence for differential alternative splicing in blood of young boys with autism spectrum disorders. *Mol Autism, 4*(1), 30. doi:10.1186/2040-2392-4-30
Suda, S., Iwata, K., Shimmura, C., Kameno, Y., Anitha, A., Thanseem, I., . . . Mori, N. (2011). Decreased expression of axon-guidance receptors in the anterior cingulate cortex in autism. *Mol Autism, 2*(1), 14. doi:10.1186/2040-2392-2-14
Talkowski, M. E., Rosenfeld, J. A., Blumenthal, I., Pillalamarri, V., Chiang, C., Heilbut, A., . . . Gusella, J. F. (2012). Sequencing chromosomal abnormalities reveals neurodevelopmental loci that confer risk across diagnostic boundaries. *Cell, 149*(3), 525-537. doi:10.1016/j.cell.2012.03.028
Tropeano, M., Ahn, J. W., Dobson, R. J., Breen, G., Rucker, J., Dixit, A., . . . Collier, D. A. (2013). Male-biased autosomal effect of 16p13.11 copy number variation in neurodevelopmental disorders. *PLoS One, 8*(4), e61365. doi:10.1371/journal.pone.0061365
Turner, T. N., Sharma, K., Oh, E. C., Liu, Y. P., Collins, R. L., Sosa, M. X., . . . Chakravarti, A. (2015). Loss of delta-catenin function in severe autism. *Nature, 520*(7545), 51-56. doi:10.1038/nature14186
Wei, H., Sun, S., Li, Y., & Yu, S. (2016). Reduced plasma levels of microtubule-associated STOP/MAP6 protein in autistic patients. *Psychiatry Res, 245*, 116-118. doi:10.1016/j.psychres.2016.08.024
