# Supplementary figures and images for: Comparative Phosphoproteomic Profiling of Type III Adenylyl Cyclase Knockout and Control, Male, and Female Mice
Source: Front Cell Neurosci. 2019 Feb 13;13:34. doi: 10.3389/fncel.2019.00034 (PMC6381875; doi:10.3389/fncel.2019.00034)

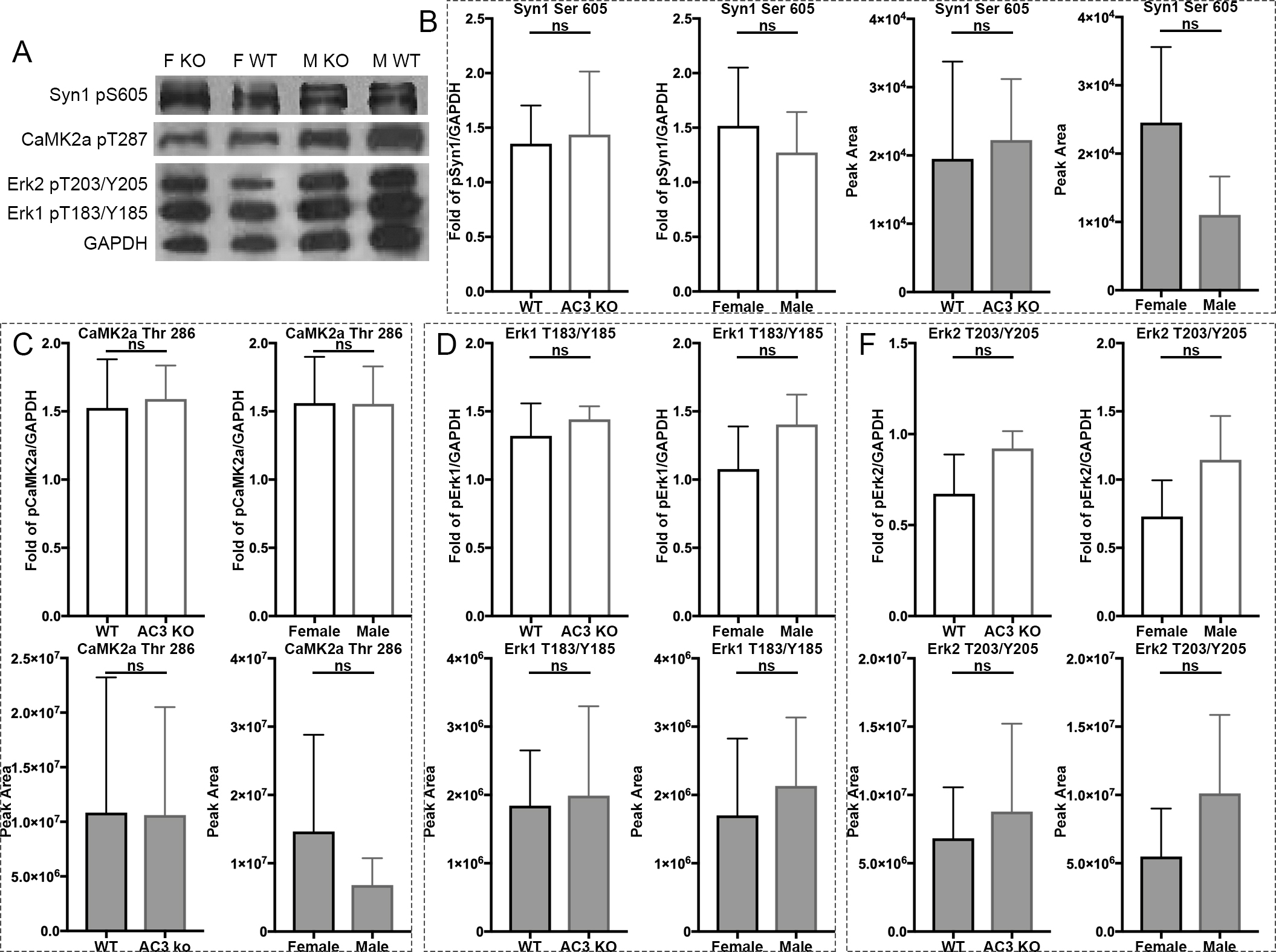

Supplement: Supplementary file 6 [file Image_1.JPEG]

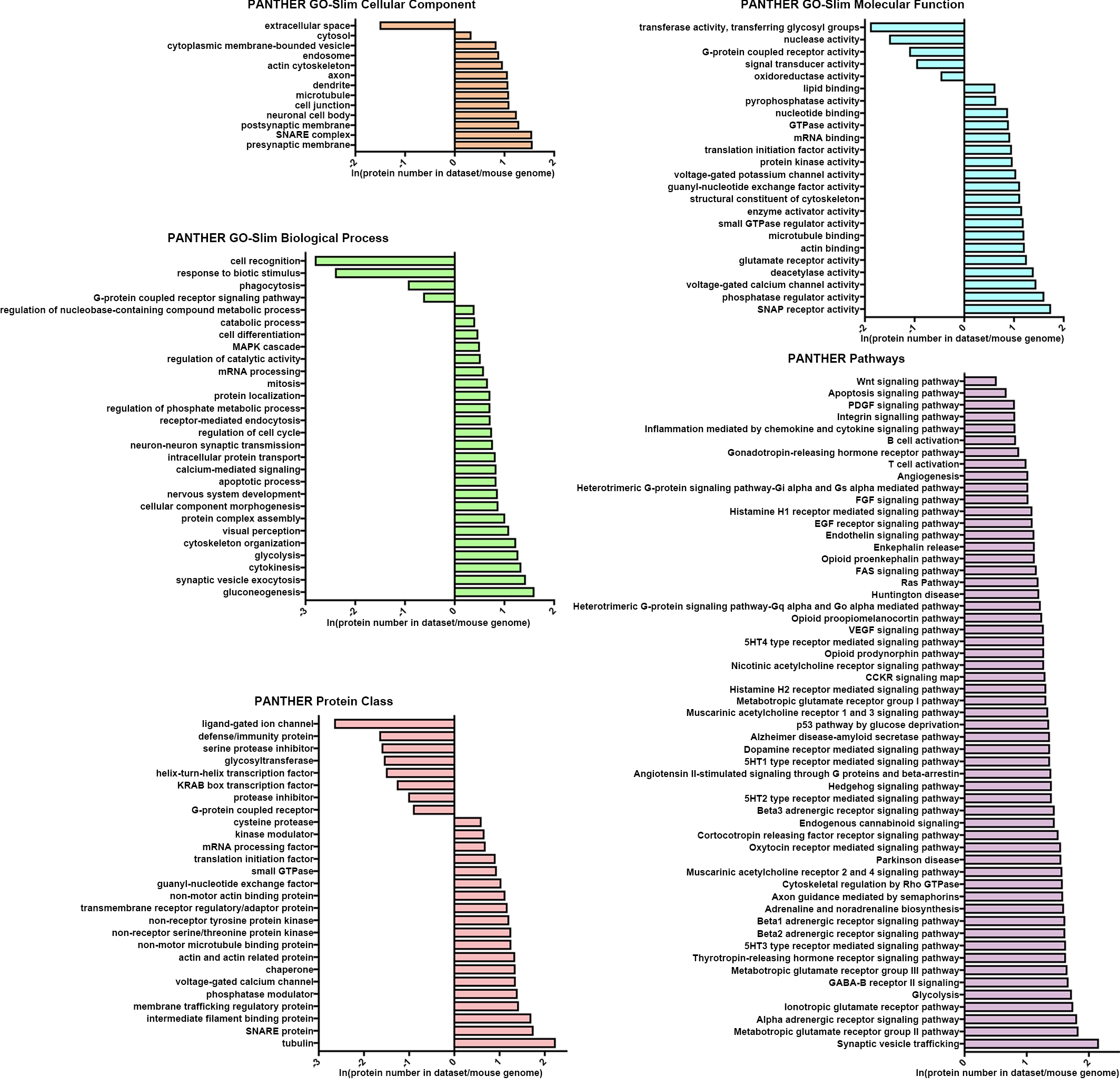

Supplement: Supplementary file 7 [file Image_2.JPEG]

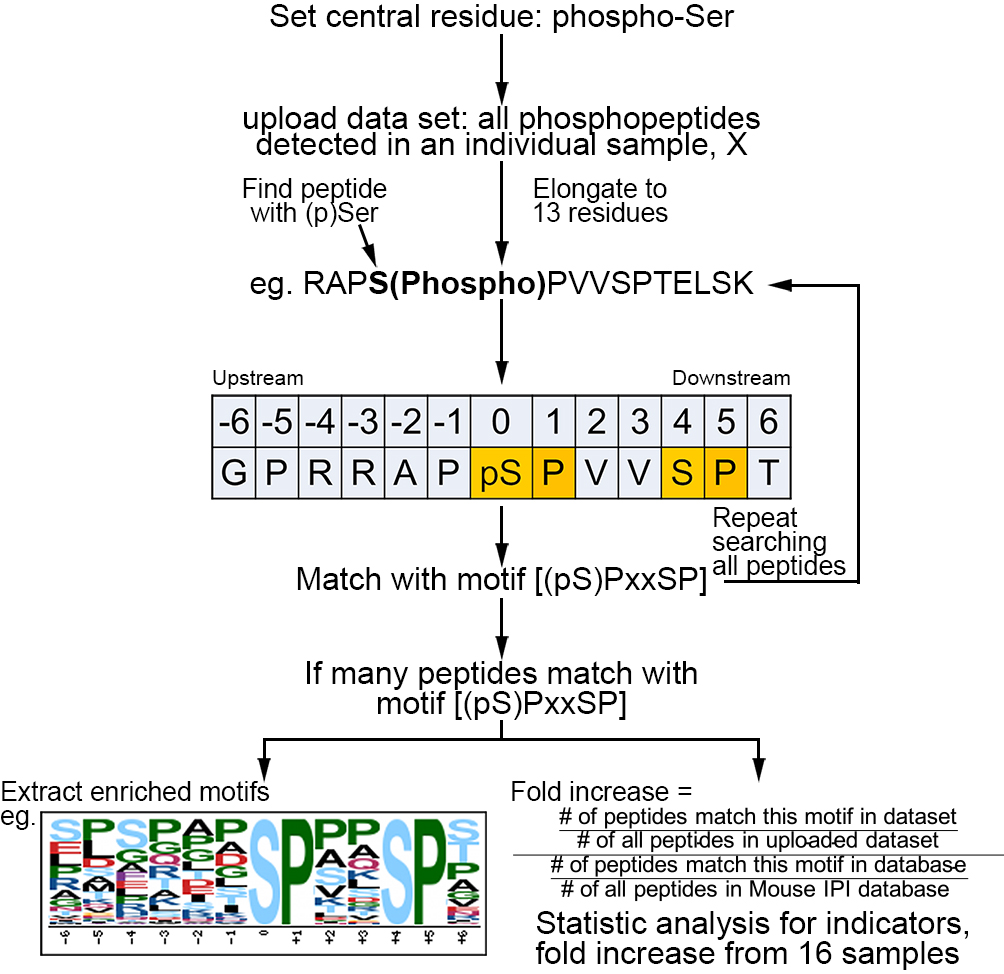

Supplement: Supplementary file 8 [file Image_3.JPEG]

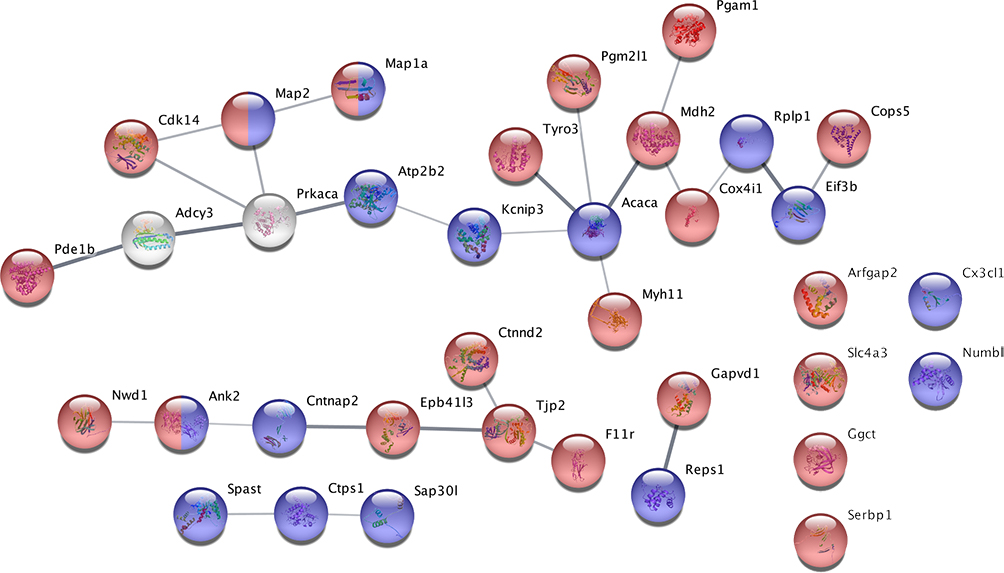

Supplement: Supplementary file 9 [file Image_4.JPEG]
